# Contrasting temperature trends across the ice-free part of Greenland

**DOI:** 10.1038/s41598-018-19992-w

**Published:** 2018-01-25

**Authors:** Andreas Westergaard-Nielsen, Mojtaba Karami, Birger Ulf Hansen, Sebastian Westermann, Bo Elberling

**Affiliations:** 1 Center for Permafrost (CENPERM), Department of Geosciences and Natural Resource Management (IGN), University of Copenhagen, Øster Voldgade 10, 1350 Copenhagen K, Denmark; 2 Department of Geosciences, University of Oslo, P.O. Box 1047, Blindern, 0316 Oslo, Norway

## Abstract

Temperature changes in the Arctic have notable impacts on ecosystem structure and functioning, on soil carbon dynamics, and on the stability of permafrost, thus affecting ecosystem functions and putting man-built infrastructure at risk. Future warming in the Arctic could accelerate important feedbacks in permafrost degradation processes. Therefore it is important to map vulnerable areas most likely to be impacted by temperature changes and at higher risk of degradation, particularly near communities, to assist adaptation to climate change. Currently, these areas are poorly assessed, especially in Greenland. Here we quantify trends in satellite-derived land surface temperatures and modelled air temperatures, validated against observations, across the entire ice-free Greenland. Focus is on the past 30 years, to characterize significant changes and potentially vulnerable regions at a 1 km resolution. We show that recent temperature trends in Greenland vary significantly between seasons and regions and that data with resolutions down to single km^2^ are critical to map temperature changes for guidance of further local studies and decision-making. Only a fraction of the ice-free Greenland seems vulnerable due to warming when analyzing year 2001–2015, but the most pronounced changes are found in the most populated parts of Greenland. As Greenland represents important gradients of north/south coast/inland/distance to large ice sheets, the conclusions are also relevant in an upscaling to greater Arctic areas.

## Introduction

Temperature changes in the Arctic influence ecosystems^1^ and ecosystem functions such as permafrost-dependent infrastructure^2,3^ and carbon stocks^4–6^. So far, mean annual mean air temperatures (MAAT) have been used to define key ecosystem variables such as the spatial distribution of permafrost^7^. Changes in annual mean air temperature may, however, be less appropriate to assess ecological changes than other parameters better reflecting growing season dynamics and thawing at the top of permafrost^8^. For instance, land surface temperatures (LST) can be of higher significance for biological processes near the Earth’s surface^9^, and for the ground thermal regime if transformed to ground surface temperature equivalents^10^. Moreover, monthly or seasonal trends in air temperature or LST are considered more relevant to assess site-specific conditions than annual averages. In combination with snow cover, topography, and drainage conditions, area-specific temperatures control the energy budget, and high spatiotemporal resolution allows for local assessments capturing the timing of changes and whether these occur at a time of year when the ecosystem impact is direct.

Coverage of air temperature stations in the Arctic is scattered and biased towards near-coastal locations, which further limits the effective spatial resolution and distribution of temperature records. Consequently, for a mountainous land mass such as Greenland, insights into the spatial variability in temperature changes based on measurements in the past decades are lacking. This study is based on satellite derived spatially distributed land surface temperatures (LST), modelled air temperatures and permafrost mapping. We quantify area-specific changes in near-surface temperature across the ice-free parts of Greenland. Time series of spatially distributed LST have increasingly been implemented in assessments of satellite sensor performance and general temperature patterns^11,12^, as well as constituting a driving parameter for regional permafrost mapping^13^.

### Mean annual air temperatures

Modelled monthly mean air temperatures 2 m above terrain, with a 5 km spatial resolution, are available from the regional climate model MAR (v. 3.5.2), forced with ERA Interim^14^. Here we analyze MAR-temperatures from the two periods 1986–2015 and 2001–2015, validated against 45 Danish Meteorological Institute (DMI) met-stations, evenly distributed across the latitudinal and longitudinal gradients of the ice-free Greenland. The validation is successfully shown as a highly significant linear fit (Fig. S1). This climate normal (30 years) is used to quantify robust mean annual air temperatures which has, as one application, been used to define the corresponding spatial distribution of permafrost zones^15–17^ (Fig. S2). Figure S2 suggests that all permafrost zones (as previously defined in refs^15–17^) are present in Greenland. A translation of air temperature into permafrost zones is not trivial, and future work should be oriented towards implementing the thermal offsets resulting from vegetation canopies during summer, and snow during winter (typically in the range of −2 to 0 °C)^18^. However, it is the first high resolution map showing the potential variability of permafrost distribution from outer to inner parts of fjord systems, as well as differences from East to West Greenland. Differences from East to West in the southern parts of Greenland are mainly due to the influence of warmer sea currents in the West^19^. The spatial distribution of the mean air temperature over 30 years shows a limited latitudinal range in July temperatures of −5 to 13 °C. By contrast, winter conditions range from −40 to −5 °C, and there are pronounced latitudinal variations in the freezing and growing degree days across Greenland (Fig. S3).

### Recently observed land surface temperatures

**Figure 1:**
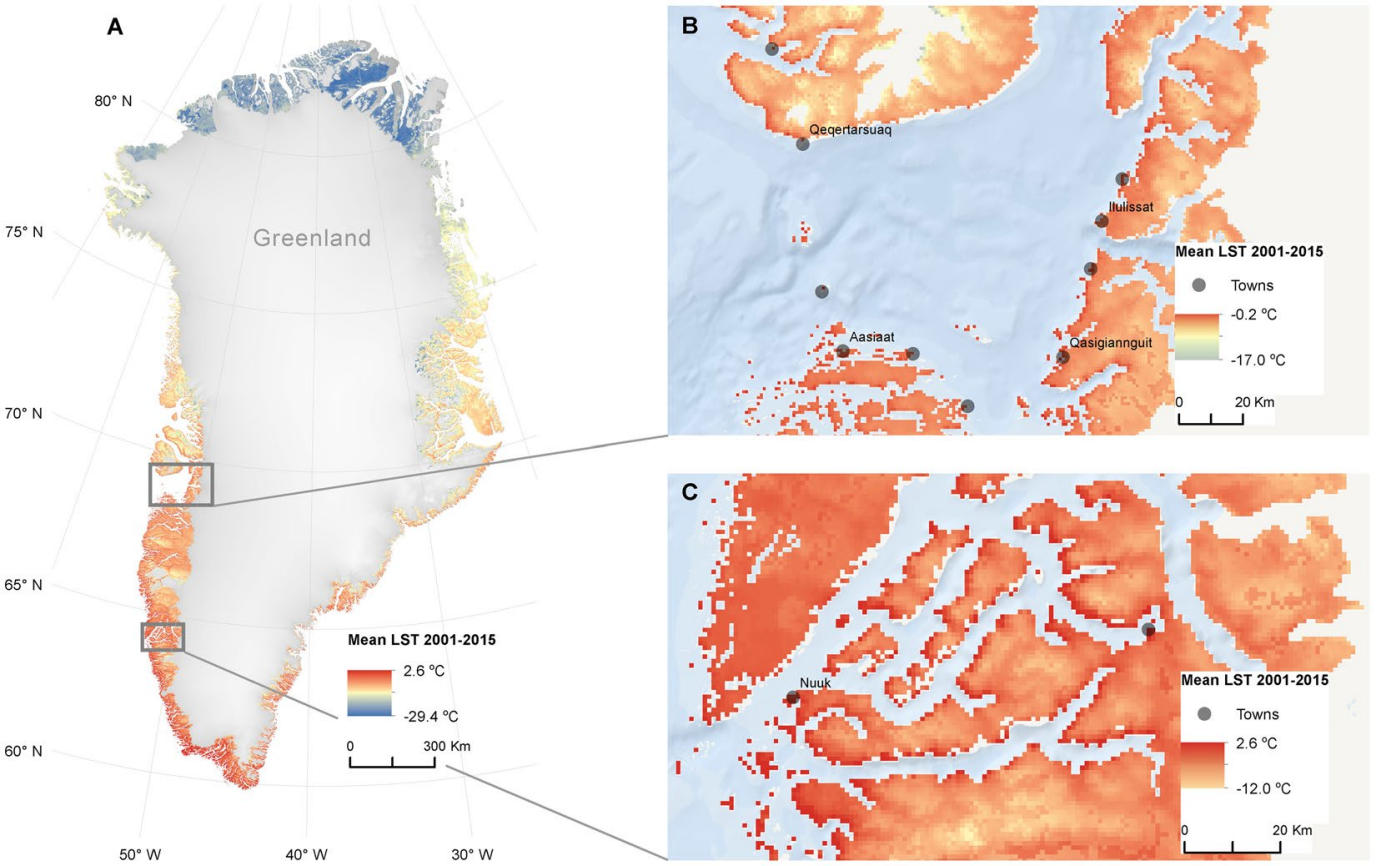
(**A**) Mean annual land surface temperature (LST) 2001–2015 from MODIS data at 1 km resolution, corrected for data gaps due to clouds. (**B**) Subset at 69 °N, illustrating elevational gradients (e.g. Northeast of Qeqertarsuaq) and coast-inland gradients (e.g. from Ilulissat and eastwards). (**C**) Subset at 64 °N of the fiord system around the capitol, Nuuk. The maps are made using ArcMap 10.3.

**Figure 2:**
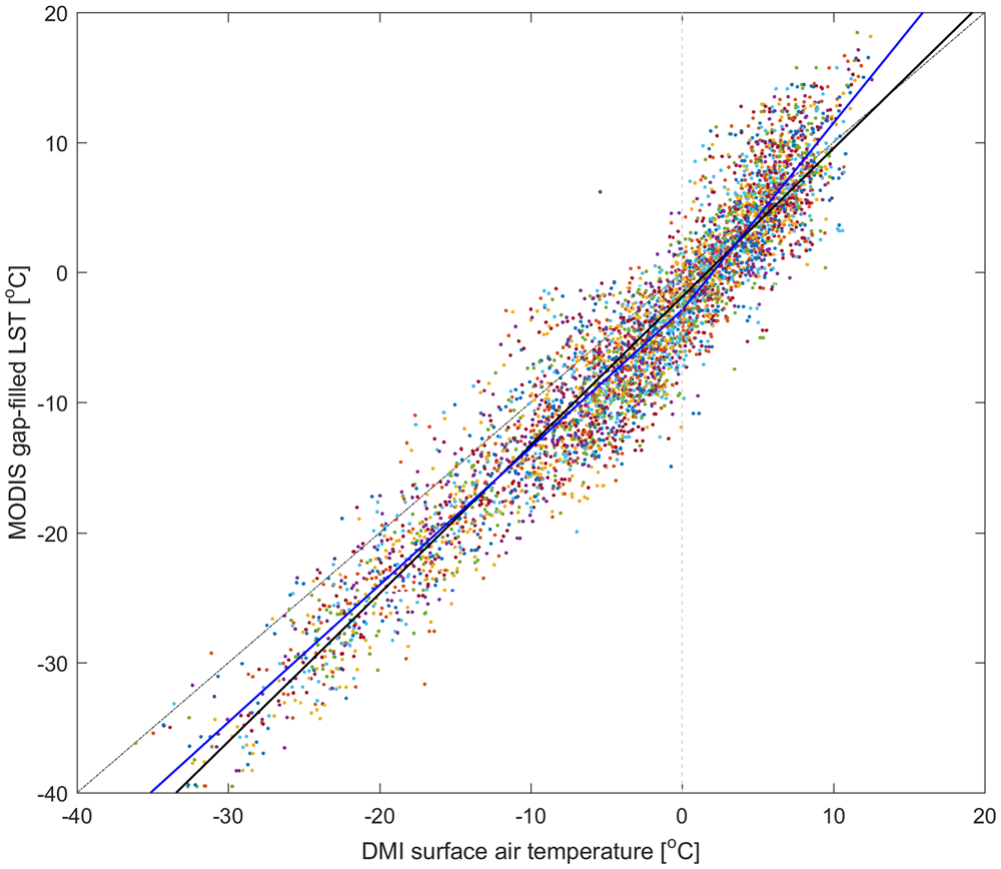
Monthly averages of gap-filled MODIS land surface temperatures (LST) and observed air temperatures at Danish Meteorological Institute (DMI) stations for the period 2001–2014. The dashed gray line displays the 1:1 relation. The solid black line is a highly significant ordinary least squares (OLS) linear regression function (p < 0.001, t-test; slope = 1.14; intersect = −1.87; RMSE = 3.33). The OLS function explains 91% of the variation (R^2^ = 0.91). The solid blue line is a stepwise OLS function, with a knot at 0 °C air temperature (dashed vertical line) (p < 0.001, t-test; knots(x) = [−40, 0, 20]; coefficients(y) = [−45.14; −2.90; 25.88]; RMSE = 3.21). Both functions illustrate the quality of MODIS LST data as a proxy for air temperatures in complex terrains. Each scatter-plot color refers to single DMI stations, illustrating an even distribution of temperatures across the stations.

The performance, availability, and operational period of the MODIS sensor onboard the Terra platform, offers unique and global spatio-temporal LST-data starting from mid-2000. It allows for detailed trend analyses in otherwise inaccessible regions, with a measured dataset fully independent of air temperature stations. This has resulted in a number of studies being based on this dataset^11,13^. Recently, a method to use LST to assess ground temperatures in a larger North Atlantic region was presented, but scarcely validated for sub-regions such as Greenland^13^. Here we use a gap-filled version of MOD11A1 (following^20^), due to otherwise missing data in cloudy conditions that can cause a significant cold bias^21^. The resulting distribution of LST data across Greenland (Fig. 1) are, however, fully consistent when using a non-gap-filled dataset (see Supporting material). The dataset is validated against the same 45 DMI stations as used before. It is showing a highly significant linear relationship between MODIS LST and air temperature (Fig. 2), with no significant difference or trend in the linear function slope between years (Fig. S4). If present, a trend in the slopes could suggest sensor drift, whilst significant annual differences in the slopes could affect the trends. A stepwise regression suggests a significantly different correlation for thawing temperatures; however this has no implications for later trend analyses since these are linear.

### Temperature trends

**Figure 3:**
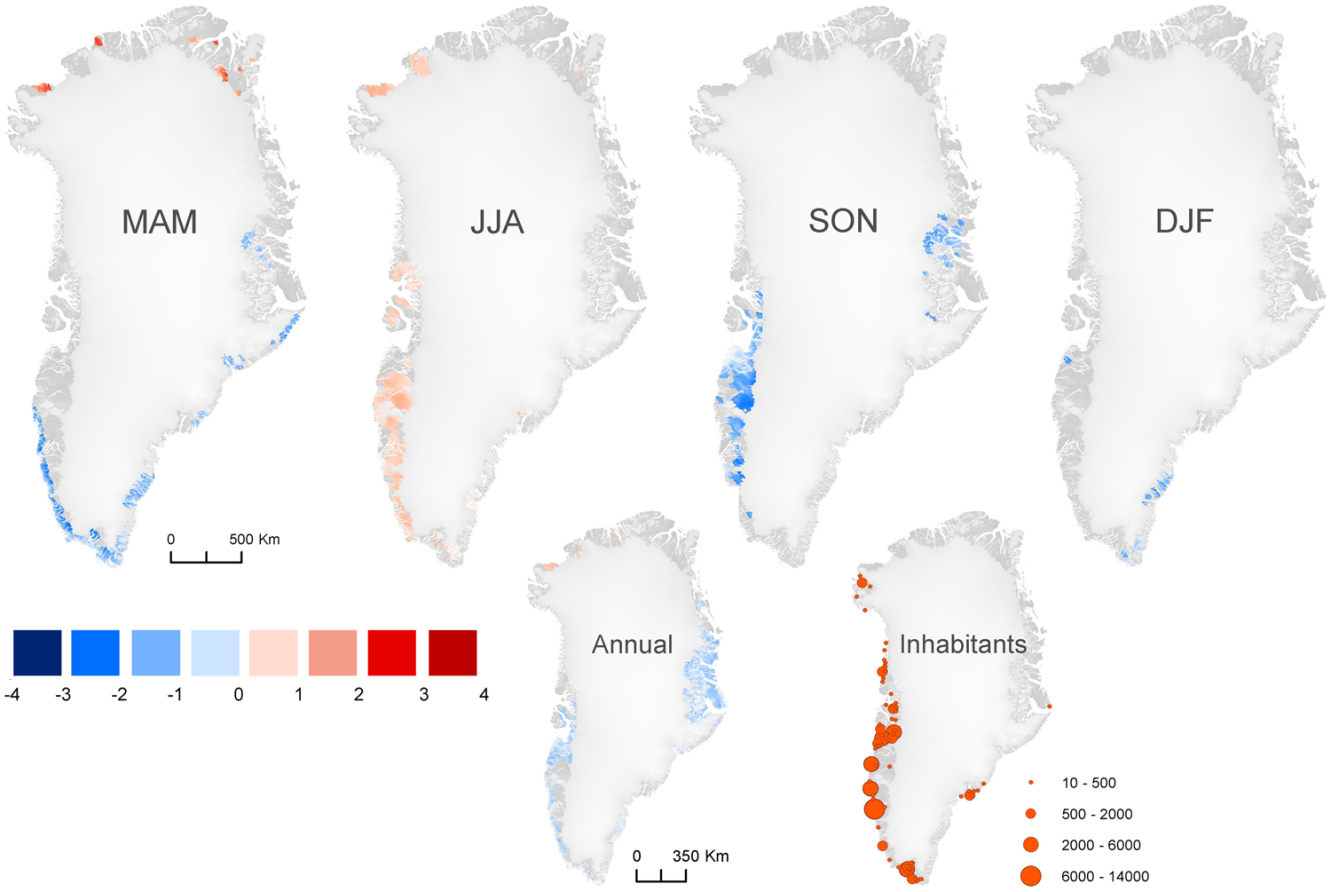
Significant changes in land surface temperatures (LST) from 2001–2015, based on gap-filled MODIS LST (seasonal Mann-Kendall and Sen slope). Seasons are shown as spring (MAM), summer (JJA), autumn (SON), and winter (DJF), as well as changes across all months (Annual). Only significant (p < 0.05) trends in temperature during 2001–2015 with more than 80 significant pixels within 20 × 20 km are shown. Significant changes are noted for up to 35% of the pixels representing the ice-free parts of Greenland. The maps are made using ArcMap 10.3.

The MAR and gap-filled MODIS LST datasets allow us to evaluate spatially distributed temperature averages and quantify temporal trends by applying the non-parametric monotonic Seasonal Mann-Kendall test to pixels along the time dimension. Here we use monthly averages to mitigate noise^22^. The analyses result in maps of mean temperatures, and linear Theil Sen-slope coefficients and p-values, respectively (Figs 1,3, S3, S5–S7). Trends in LST could in theory be resulting from land-use changes, however, Greenland is a pristine environment with only local anthropogenic impact, and Fig. 2 supports the strong relationship with air temperature which would be less influenced by local changes in land-use. The trends are filtered to include values between the 5^th^ and 95^th^ percentiles only, with a statistical significance (p-value, Mann-Kendall test) lower than 0.05. Areas with statistically significant temperature trends are filtered with a moving 20 × 20 pixels window, in which only areas with more than 80 significant pixels within the window are accepted as having a temperature change (corresponding to 20% of the analyzed window area). Following the filtering, the mean trend is computed within the moving window. The filtering results in more robust and conservative quantifications of change. Figure 3 shows that on an annual basis, less than 36% of the ice-free Greenland has experienced a significant trend and, if any, a cooling is observed during the last 15 years (<0.15 °C change per year). But by splitting the data set in four seasons, we find a significant summer warming in West Greenland while a significant spring cooling is seen in South Greenland and a marked autumn cooling is wide-spread in Southwest and mid-Greenland. A similar picture is seen if the same analysis is made based on MAR data from 2001–2015, i.e., limited spatial extent of significant changes on an annual basis (<0.17 °C change per year) while significant regional trends are observed within different seasons (Fig. S5). If the analysis is extended to 1986–2015, an overall warming trend is noted (Fig. S6). This highlights the importance of a warming dominating the 1990’s while more fluctuating conditions and complex temporal and spatial trends have dominated since 2001. The trend analyses per individual months (Fig. S7A,B) highlight the importance of shorter time intervals, to avoid averaging warming and cooling trends out over time and regions, and to be able to discuss ecosystem functions such as permafrost stability when little or no change is observed at annual time steps. Furthermore, these trends help to discuss and understand drivers for changes, one example being the significant autumn (Sep., Oct., and Nov.) cooling, which is observed in different regions in Greenland, depending on the specific month. These shifts are probably related to ocean currents and sea ice dynamics^23^, local wind regimes and topography, and regional teleconnection phenomena^19^. The above analyses show trends on regional (10–100 km) and even local scales (0.5–10 km) which in the current state, and if projected into the future, would be relevant for planning and resource management in Greenland. This approach seems furthermore relevant in the entire ice-free Arctic.

### Observed temperature trends in a future context

Using recorded air temperature from Nuuk, Ilulissat, and Tasilaq since 1895, significant correlations between seasonal temperatures and Greenland Blocking Index^19^ (GBI) anomalies can be noted, with the strongest link being in West Greenland during spring and winter (Table S1). The general fluctuations in GBI in the same period suggest a negative phase in the 1990s where we observe a warming trend, followed by a positive phase during 2000–2015, where we observe a period with stable or cooler temperatures. Following this relationship between observed temperatures and GBI anomalies, we may be entering a new warming period, i.e., the GBI is moving towards a higher frequency of negative anomalies (Fig. S8). This effect would be masked if future projections are employed that do not represent the regional signals, and the true timing of a temperature change could shift^24^. As a result, a future scenario that combines both anthropogenic climate forcing (including land use changes and greenhouse gas emissions) and periods with natural warming may increase permafrost thaw more than suggested by this study. When anthropogenic and natural warming are synchronized again, as observed in 1990–2000, it can be expected to result in rapid thaw of the discontinuous permafrost and greatly reduce the response time for adaptation measures.

### High-resolution temperature data as a request for ecosystem service evaluation

Air- and surface temperature trends control to a great extent the growing season dynamics^25^, active layer thickness, the distribution and vulnerability of permafrost^26^ and biochemical soil processes^27^. The stability of these processes and robust projections are important for societies depending on these as ecosystem services. Stable permafrost conditions in particular are important to support buildings and infrastructure, reduce risk of erosion, flooding, landslides and collapsing, allow for local cooling for storage, and protect archaeological remains. Furthermore, stable conditions help to preserve the large carbon stocks in permafrost soils^6,28^. Areas currently dominated by warm permafrost which coincide with areas subjected to climate warming, are considered the most vulnerable regions. Pin-pointing the specific slopes, buildings etc. at risk requires additional *in-situ* work including surveys of ground ice content and surficial geology, which will have decisive effects locally^29^. Rather the presented results call for a future use of satellite-based independent temperature datasets as an alternative to sparsely distributed meteorological stations with a bias towards coastal areas. In Greenland generally, areas subjected to unstable permafrost conditions are the ones characterized as discontinuous permafrost (Fig. S2), where future warming will markedly disintegrate the permafrost. This vulnerable permafrost is found in West Greenland (Fig. S2) where most people are living today (Fig. 3) and in areas where infrastructure such as airports and harbors are not all located on bedrock. Whilst a lack of marked warming trends during the last 15 years can restrain permafrost thaw, it should not be over-interpreted, since we cannot differentiate between anthropogenic forcing and natural fluctuations in this study.

### From this study we conclude

1. Recent warming varies significantly between seasons and regions in Greenland. High-resolution temperature data with respect to space and time has several applications for instance as a prerequisite to analyze the current presence of permafrost and likely levels of change, at a scale relevant for the society. Remote sensing products provide such data and have successfully been validated against observations.
2. Warming trends observed from 1986–2016 across the ice-free Greenland is mainly related to warming in the 1990’s. The most recent and detailed trends based on MODIS (2001–2015) shows contrasting trends across Greenland, and if any general trend it is mostly a cooling. The MODIS dataset provides a unique detailed picture of spatiotemporally distributed changes during the last 15 years.
3. Using this high-resolution data set, this study discusses the implications for growing season dynamics, active layer dynamics and potential permafrost degradation during the last 15 years in relation to the location of Greenlandic settlements for the first time. The analysis illustrates the need for high-resolution data and projections, to assess temperature variations in locations far from *in-situ* temperature observations.
4. A summer warming is shown in Greenland, particularly in parts of the more populated areas. Therefore, future quantification of soil temperatures, active layer development and permafrost thawing rates are important, and research in this regard should be oriented towards development and validation of transient climate-permafrost-ecosystem modelling.

## Methods

### Processing of MODIS land surface temperatures

Land surface temperatures (LST) were derived from the Moderate Resolution Imaging Spectroradiometer (MODIS) sensor on-board the Terra satellite platform. The precision aim of MODIS LST measurements is ≤1 K^30^, however studies from the Arctic region has suggested a cold bias error of up to 3 K due to clouds contaminating the signal^21^. Here we consequently use cloud-free data only which are gap-filled based on empirical relationships between estimates of net daily shortwave radiation and LST, as described in ref.^20^. Terra overpasses central Greenland at approx. 23:30 and 15:30 GMT in ascending and descending mode respectively. This study is based on these daily overpasses, synthesized in the MOD11A1 v005 product. We used tile h15v02, h15v03, h16v00, h16v01, h16v02, h17v00, h17v01 and h17v02 to cover the entire Greenland. The tiles were quality filtered, using only cloud-free pixels with a mean emissivity error <0.04 and mean LST error <3K.

A similar but independent setup to the Terra satellite is found on the Aqua platform. Given inaccuracies resulting from a geolocation mismatch between Aqua and Terra, and the modest gains in the estimation of LST when adding Aqua data, these were not used. Moreover, Terra offers two additional complete years of data.

### Validation of LST and MAR-data with air temperature

Climate records from the Danish Meteorological Institute covering parts of or the whole analyzed period from 2001–2014 were used to build a validation function of LSTs and air temperature. Data was used from a total of 45 sites evenly distributed across the ice-free parts of Greenland. All air temperature measurements were based on dry bulb temperature at 2 m elevation above terrain level. The sample frequency varied between 6 to 24 daily observations, which we averaged to monthly mean temperature. Air temperatures were then compared to the corresponding MODIS pixels (i.e., the pixels covering the area where the climate stations were/are located) using a scatter plot, to which we fitted an ordinary least squares (OLS) linear regression, and a two-node stepwise linear regression function (Fig. 2). For positive air temperatures, the functions were significantly different (t-test, p < 0.05), however, the computed LST trends are not influenced by the choice of validation function. We found no significant difference in the OLS linear function slopes between years, within 95% confidence intervals (Fig. S4). There were no significant differences in the function slopes which could be related to distance to coast, elevation, distance to the Greenland Ice-sheet, latitude or longitude (generalized linear model, t-test). We also found a greater variation within geo-regions (DMI Technical Report 00–18) than between geo-regions (generalized linear model, t-test).

### Trend analyses

The statistical significance of all trend analyses on 1 km MODIS LST data and 5 km MAR 3.5.2. model data were computed using the non-parametric monotonic Seasonal Mann-Kendall test. The rates of change were computed with the Theil Sen slope. All trend analyses were repeated using a Generalized Linear Model to evaluate possible spatial differences, see Supporting material. Seasonal trends were based on the corresponding three monthly averages per year, in total 45 data points per pixel. Only statistically significant (p < 0.05, Mann-Kendall test) trends were accepted as changes. The ice-free part of Greenland was masked with a polygon based on recent aerial photos and high spatial resolution satellite imagery, produced and validated by Geological Survey of Denmark and Greenland (GEUS).

### Mapping

The permafrost map (Fig. S2) is based on MAAT from the MAR 3.5.2 model, computed for the climate normal period 1986–2015. The MAAT were subsequently grouped into classes corresponding to the permafrost zones “None”, “Sporadic”, “Discontinuous”, and “Continuous” The classes were consistent with the general perception of how permafrost is distributed with regards to MAAT only^15,17^. The illustrated towns and settlements in Fig. 1, including the population densities, are based on 2016-data from Greenland Statistics (www.stat.gl). Geolocation of the towns and settlements is acquired from NunaGIS (www.nunagis.gl). All maps are made using ArcMap 10.3 http://www.esri.com/en/arcgis/products/arcgis-pro/overview and all graphs using Matlab 2015b https://www.mathworks.com/products/matlab.html.

## Electronic supplementary material

Supplementary Information
