# Supplementary material for: Contrasting temperature trends across the ice-free part of Greenland
Source: Sci Rep. 2018 Jan 25;8:1586. doi: 10.1038/s41598-018-19992-w (PMC5785469; doi:10.1038/s41598-018-19992-w)
Supplement: Supplementary file 1 — Supplementary Information [file 41598_2018_19992_MOESM1_ESM.doc]

**Supporting material for**

Contrasting temperature trends across the ice-free part of Greenland

Andreas Westergaard-Nielsen^1^, Mojtaba Karami^1^, Birger Ulf Hansen^1^, Sebastian Westermann^2^ & Bo Elberling^1^*

*^1^ Center for Permafrost (CENPERM), Department of Geosciences and Natural Resource Management (IGN), University of Copenhagen, Øster Voldgade 10, 1350 Copenhagen K, Denmark*

*^2^Department of Geosciences, University of Oslo, P.O. Box 1047, Blindern, 0316 Oslo, Norway*

*Correspondence: [be@ign.ku.dk](mailto:be@ign.ku.dk)

**METHODS**

**Gap-filling of MODIS land surface temperatures**

Land surface temperature (LST) is recorded from a day-time and night-time overpass of the MODIS sensor. Both recordings are available in the MOD11A1-product, including emissivity derived from MODIS band 31 and 32. Here we first quality-filtered data by omitting data with bit 04-05, legend 11 and bit 06-07, legend 11. Secondly we gap-filled data-gaps due to clouds using a linear model derived from 1) modelled net shortwave radiation at the Earth surface, 2) one or both diurnal MODIS LST recordings. The linear model was calibrated using in-situ measurements of net shortwave radiation and LST from the Greenland Ecosystem Monitoring stations in Kobbefjord, Disko, and Zackenberg, resulting in a regression model describing in-situ LST from the shortwave radiation. The model was used instantaneously corresponding to the satellite overpass time. We developed three models corresponding to the three scenarios where MODIS data is availability for day-time, a night-time, or both. Daily net shortwave radiation at 1 km spatial resolution was computed using incoming shortwave radiation from ERA-Interim, and albedo from MCD43B3, The MODIS LSTs were subsequently gap-filled by relating the modelled radiation and albedo to the linear gap-filling model (see also^19^). All MODIS data were kept in the original sinusoidal projection during the analyses, and converted to UTM Zone 24N for the graphical presentations.

**Validation of LST and MAR-data with air temperature**

Data from the Danish Meteorological Institute was used from the following stations (using the World Meteorological Organisation numbers of the stations): 4201, 4202, 4205, 4207, 4210, 4211, 4212, 4213, 4217, 4219, 4220, 4221, 4224, 4228, 4230, 4231, 4234, 4241, 4250, 4253, 4254, 4260 (Mitt. Paamiut), 4260 (Paamiut Heliport), 4266, 4272, 4273, 4280 (Narsaq), 4280 (Narsaq Heliport), 4282, 4283 (Nanortalik), 4283 (Nanortalik Heliport), 4285, 4301, 4310, 4312, 4320, 4330, 4339, 4341, 4351, 4360, 4361, 4373, 4382, 4390 (DMI Technical Report TR 16-04). Monthly averages were derived from up to 24 daily observations. We validated the MODIS LST and modelled MAR air temperature against the measured data from the DMI stations using a linear model (Fig. 2 and S1). The use of a two-node stepwise linear function or 2^nd^ order polynomial did not improve the correlation significantly (t-test). The model fit was significantly different (99% confidence, t-test) between a linear validation model based on gap-filled LST and air temperature (1.14x – 1.87; R^2^ = 0.91; p < 0.001; RMSE = 3.33; Fig. 2), and a linear model based on non-gap-filled LST and air temperature (1.17x – 1.55; R^2^ = 0.89; p < 0.001; RMSE = 3.79), i.e., the gap-filled dataset is less biased with a slope closer to one, and with lower residuals.

Modelled atmospheric monthly mean temperatures at 5 km spatial resolution was obtained from the regional climate model MAR (v. 3.5.2), forced with ERA Interim, and fully coupled to the CROCUS snow model^14^.

**Filtering of trend analyses**

The 5^th^ and 95^th^ percentile within the pixels showing a significant trend over time in the ice-free region, were filtered out in the gap-filled MODIS dataset to remove outliers. The maps with significant changes were filtered with a moving window of 20x20 pixels, where only pixels in windows with more than 80 significant pixels were accepted, corresponding to 20% of the ice-free landmass within the window.

**Figures for supporting material (contrasting climate trends across the ice-free part of Greenland)**


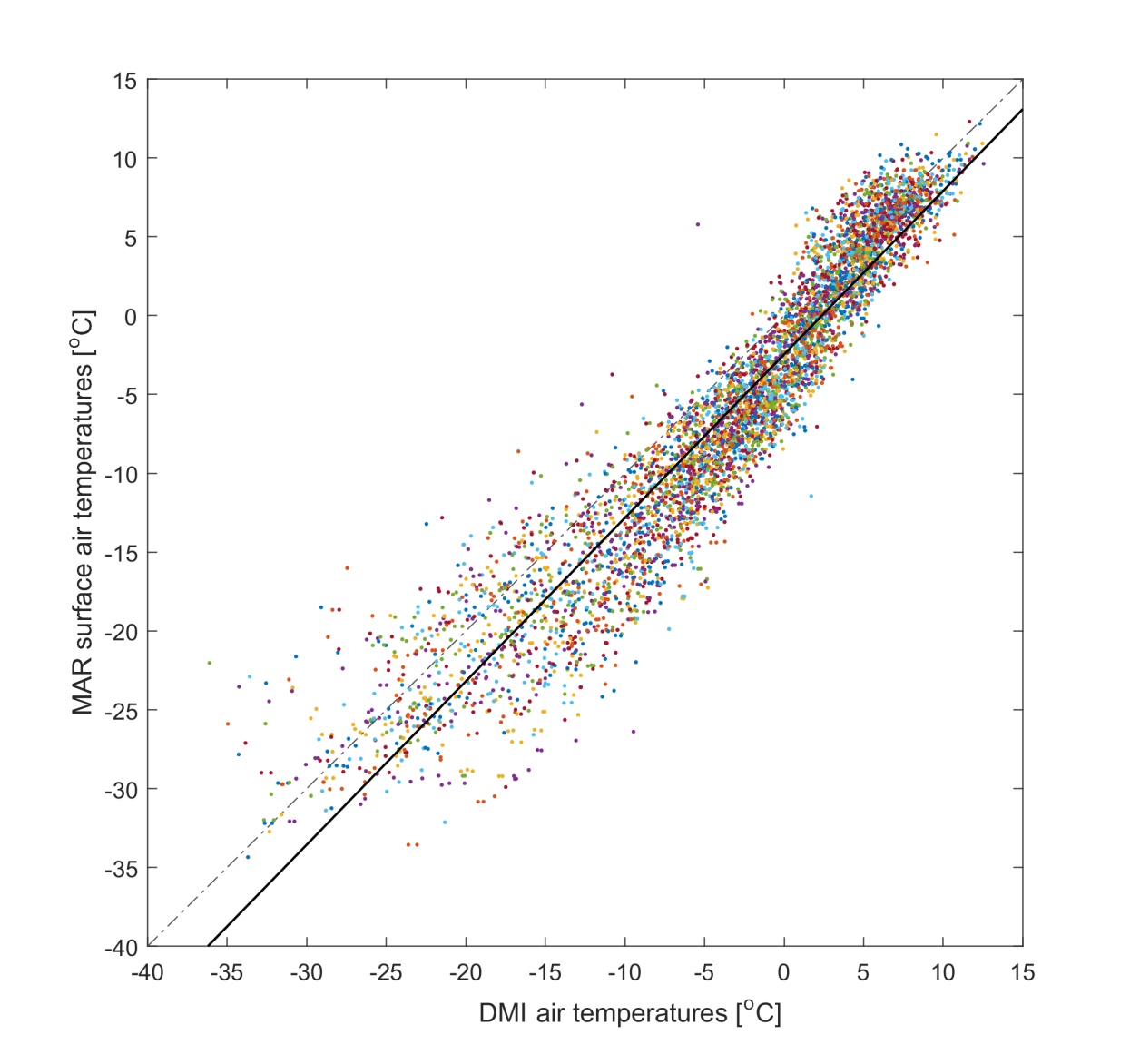

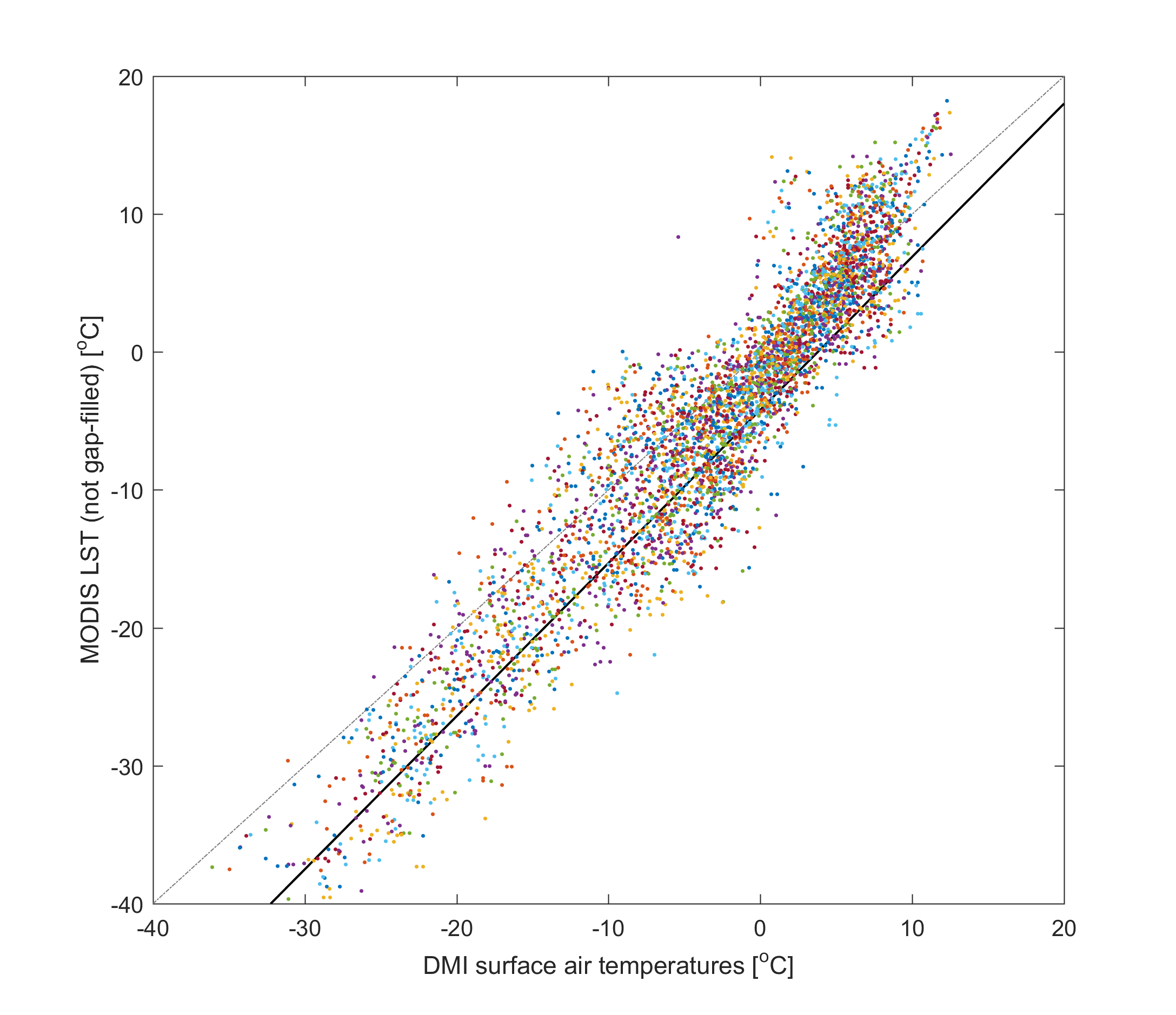


A B

Supporting figure S1: A) Correlation analysis between air temperatures (2m above terrain) from MAR v3.5.2 at 5 km resolution, forced with ERA Interim, and DMI stations. The solid black line shows an ordinary least squares linear regression function (1.04x – 2.45; R^2^ = 0.90; p < 0.001; RMSE = 3.07). B) Correlation between MODIS MOD11A1 LST (not gap-filled with cloud-correction) and DMI stations (1.17x – 1.55; R^2^ = 0.89; p < 0.001; RMSE = 3.79). The dashed lines show the 1:1 relation. Each unique color in the scatterplots reflects individual DMI stations.


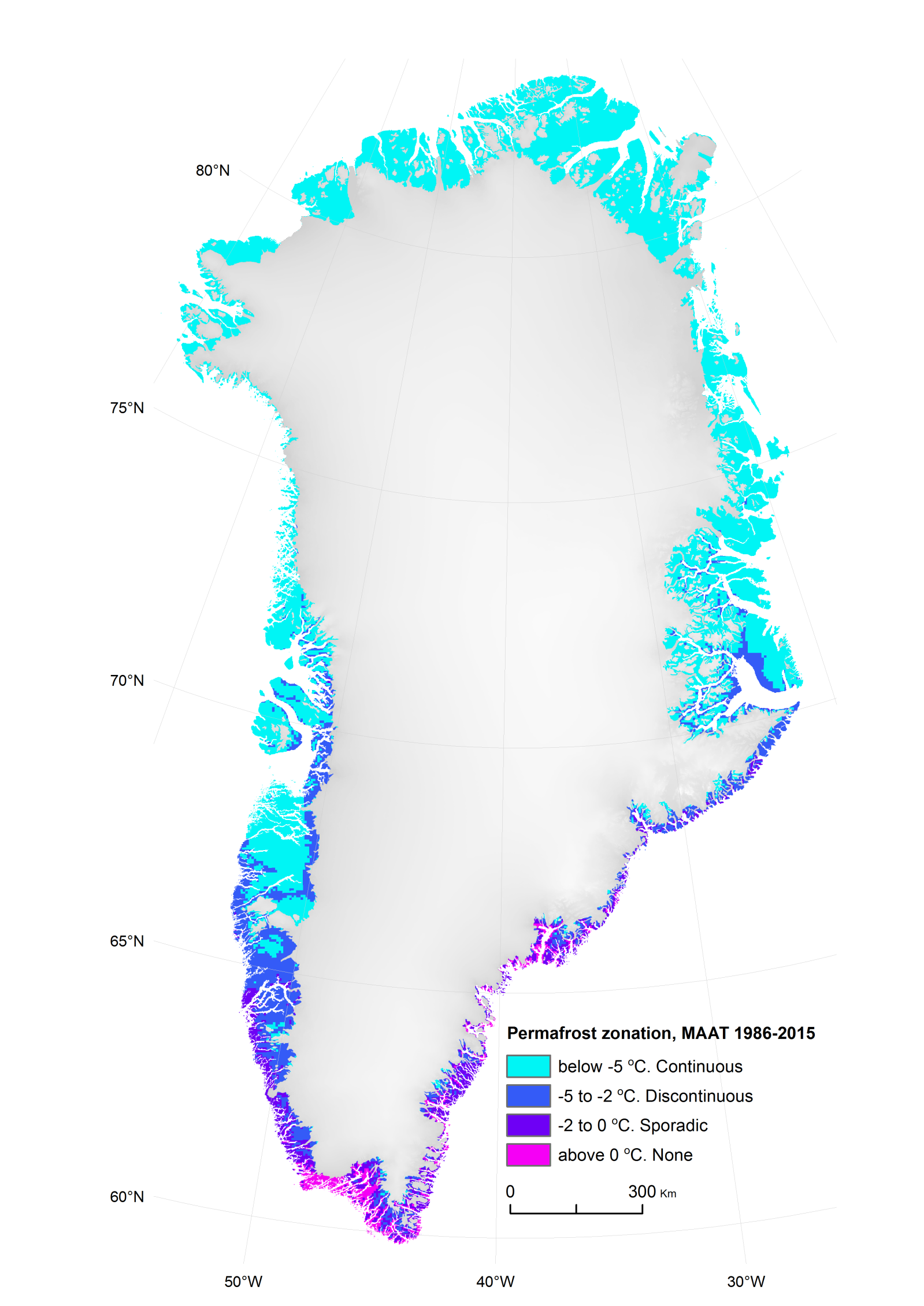


See figure S2B

Supporting figure S2A: General spatial distribution of permafrost types in ice-free Greenland. The classes are based on mean annual air temperatures (MAAT) from MAR v.3.5.2, covering the climate normal 1986-2015. The 5 km spatial resolution captures general gradients from coast to inland, latitudinal, and East to West. The maps are made using ArcMap 10.3.


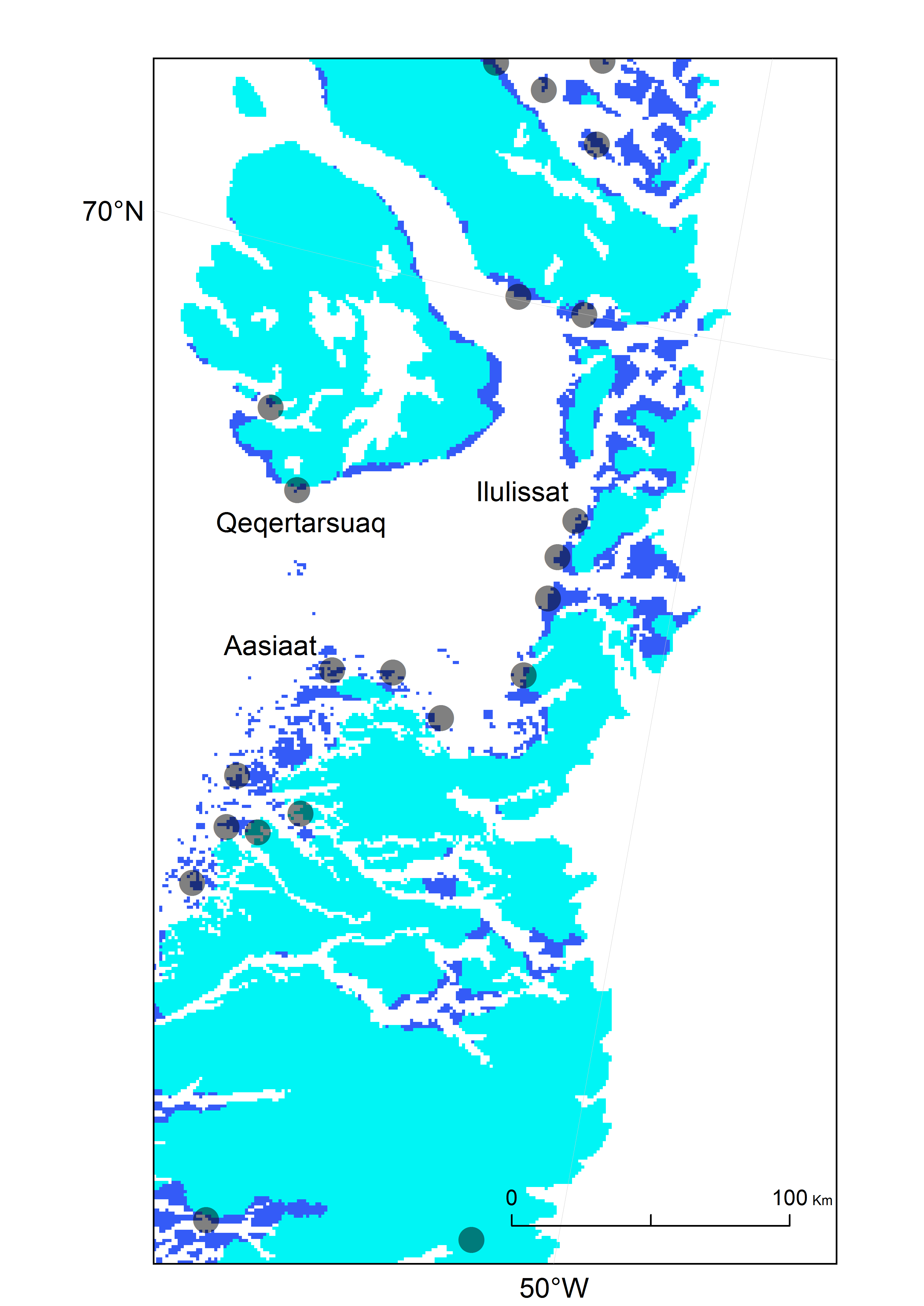


Supporting figure S2B: Downscaled permafrost zonation map based on 1km MAAT from MAR 3.6.4., which has been adjusted for topography. The map illustrates how towns are located near the coast, often at elevations below 200 m.asl. Consequently, higher temperatures occur, resulting in higher likelihood for discontinuous permafrost. The maps are made using ArcMap 10.3.


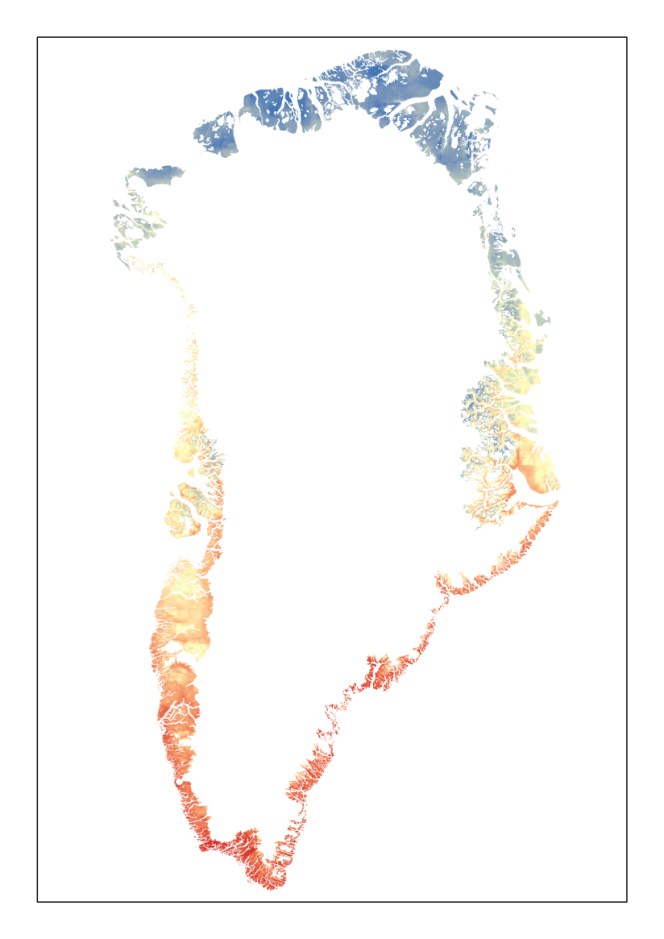

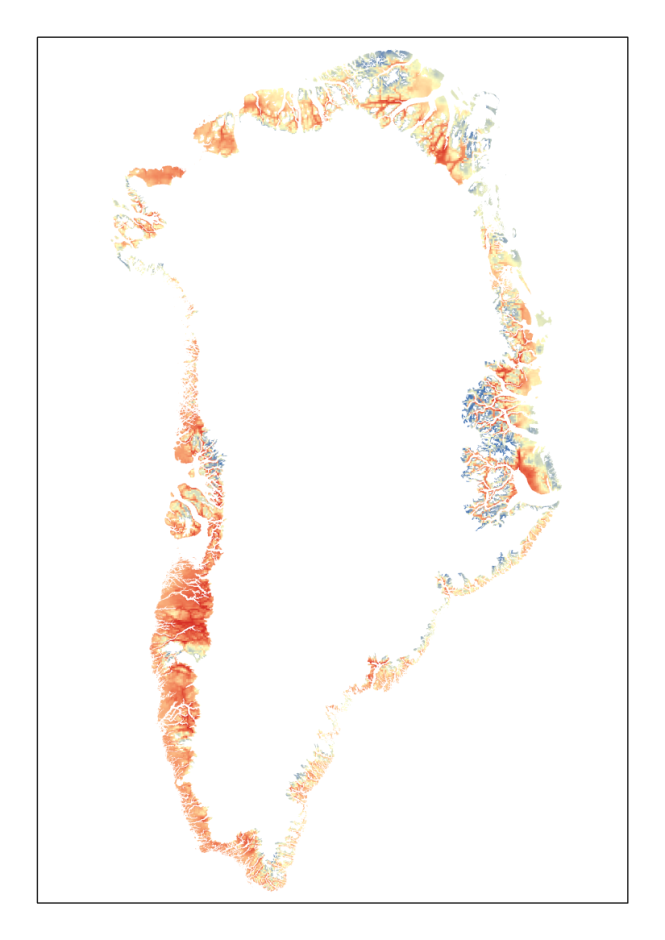

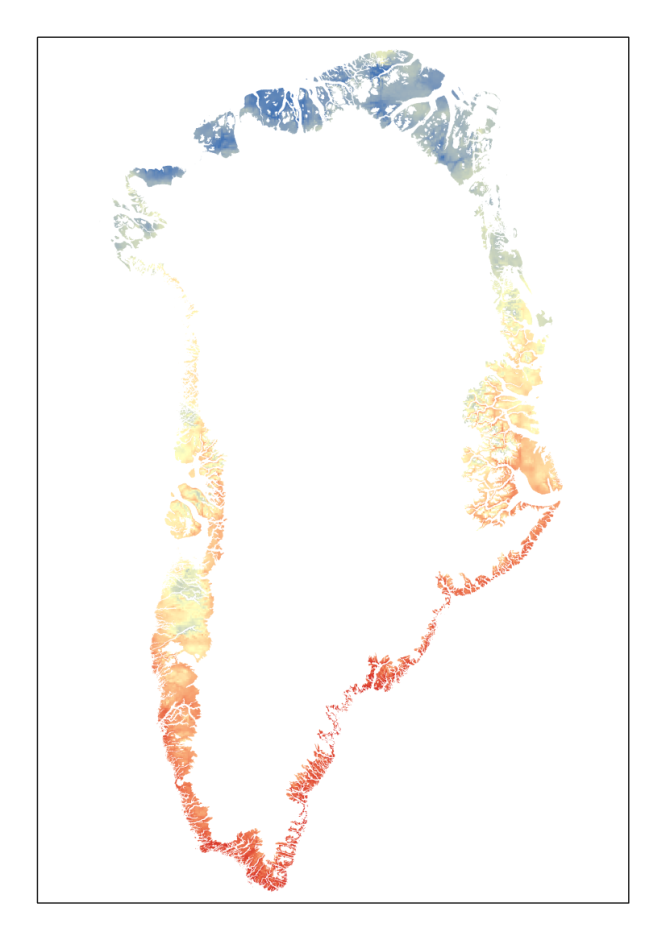


Annual mean July mean January mean


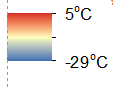

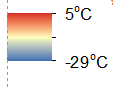

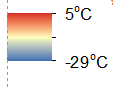


13^o^C

-5 ^o^C

-5^o^C

-5^o^C

-40^o^C

3^o^C

-22^o^C


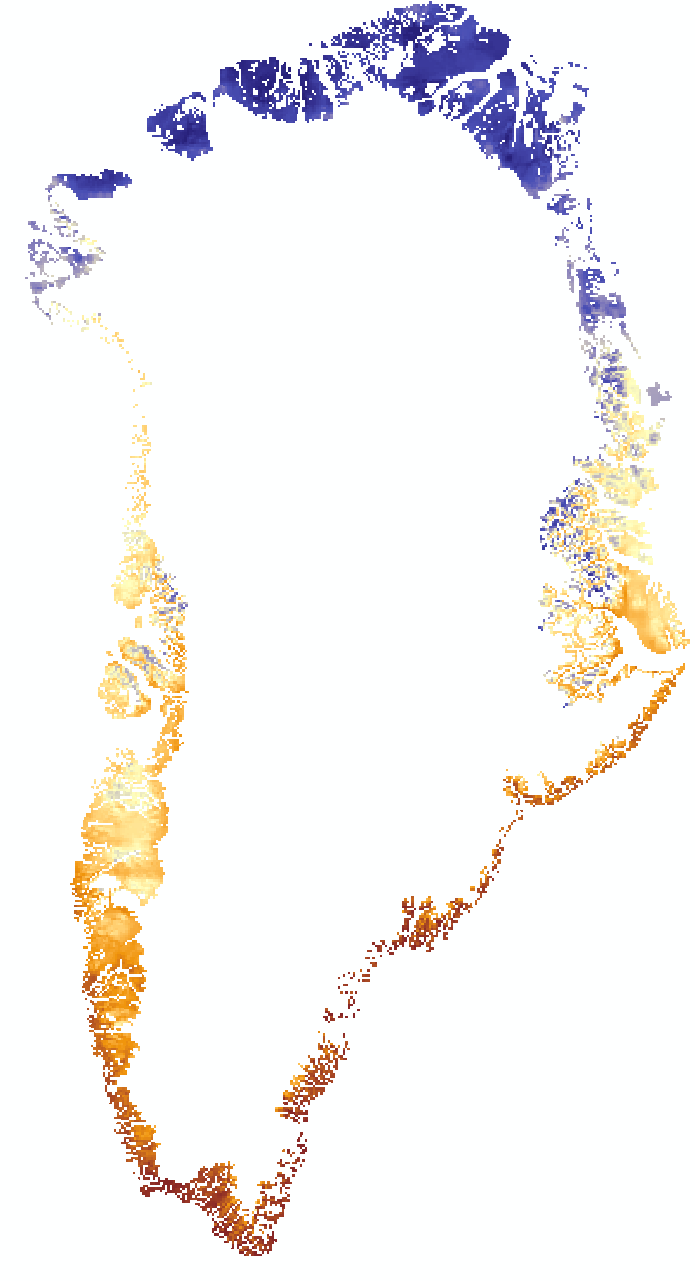

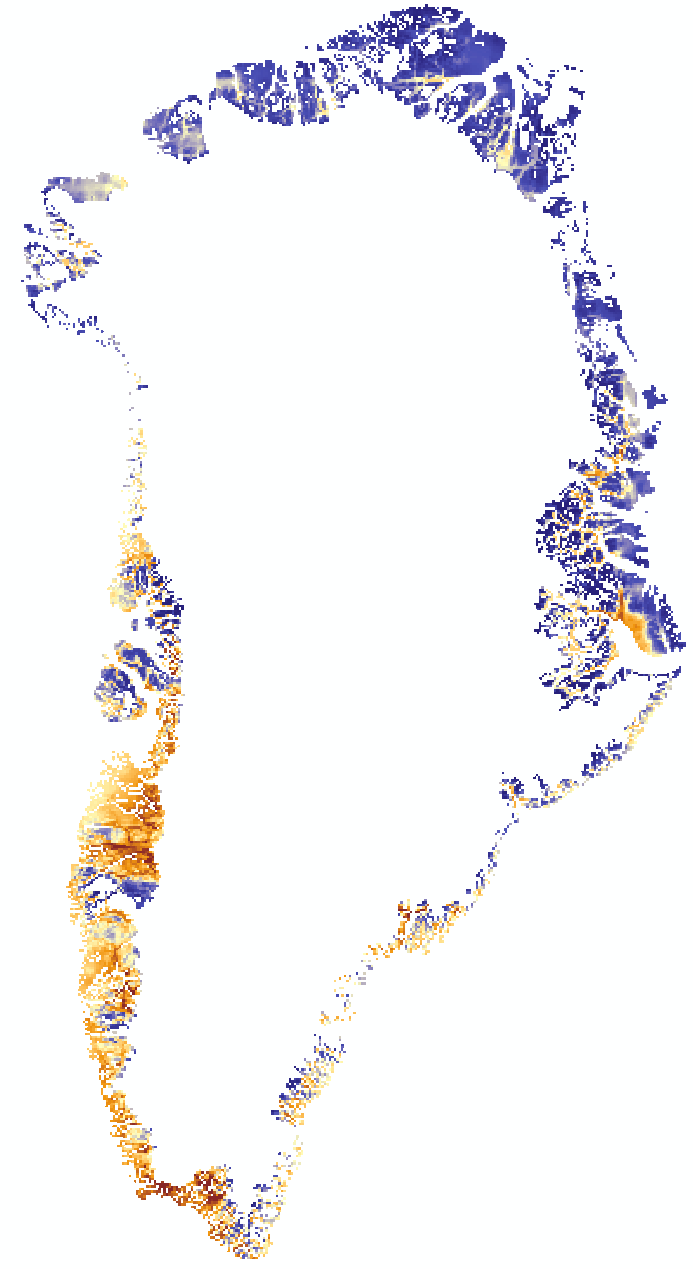


Supporting figure S3: Air temperatures from MAR v3.5.2 at 5 km resolution, forced with ERA Interim, from 1986-2015. Relatively low temperature ranges from North to South are seen during July, whereas annual mean and January temperatures exhibit a larger range. Freezing and thawing degree days are computed as the accumulated mean monthly temperatures below and above 0^o^C per year, respectively. The maps are made using ArcMap 10.3.

Mean freezing degree days

Mean thawing degree days


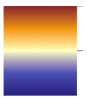

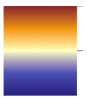


76.3

0.0

-54.5

-553.0


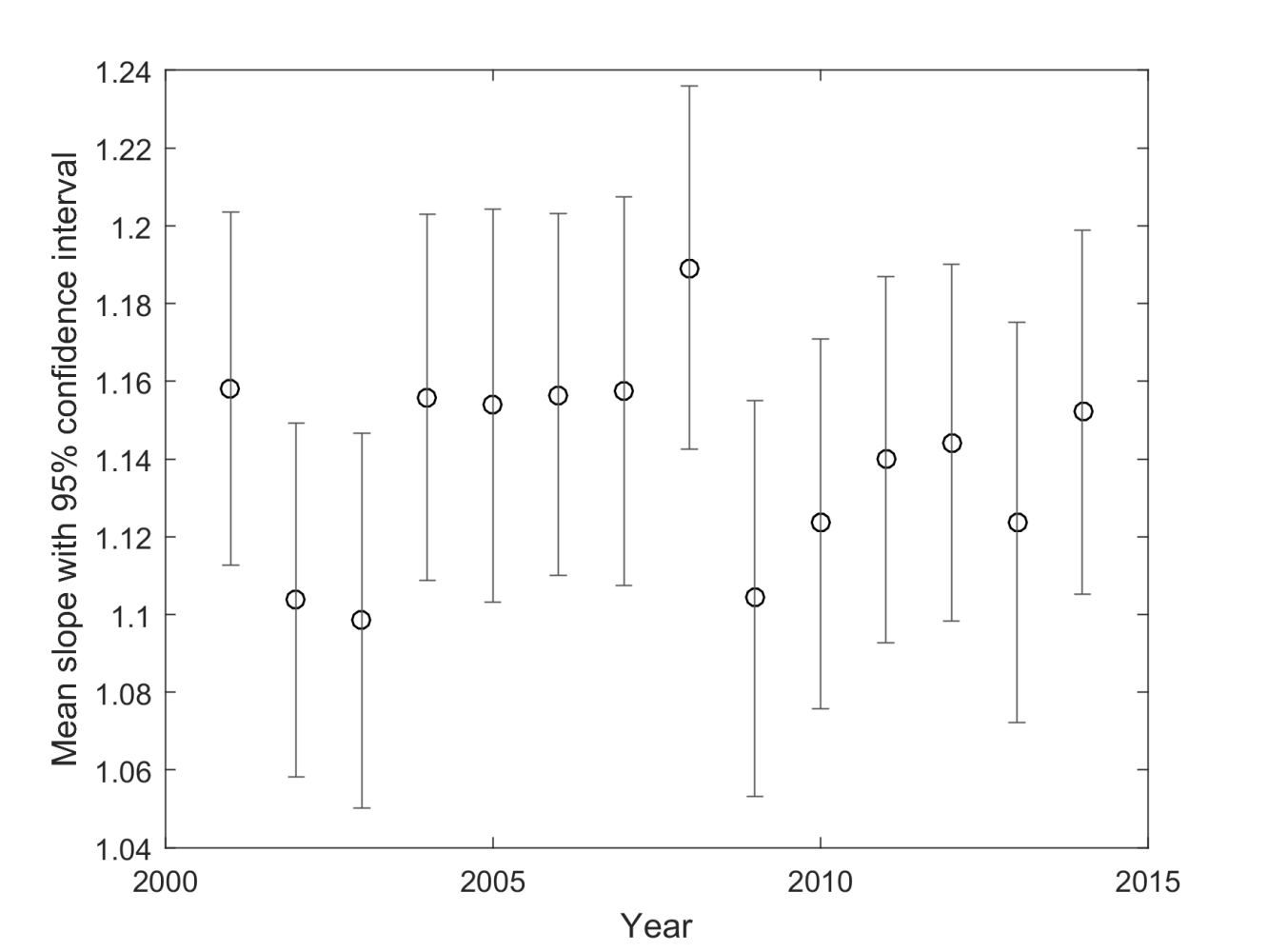


Supporting figure S4: Variations in mean slope (bars indicate the 95% confidence intervals) of the linear function between DMI monthly air temperature and gap-filled monthly LST, from 2001 to 2014. There is no trend in the mean slopes over time, and the slopes are not statistically significantly different (overlap test) between years. Mean slope for all years is 1.14.


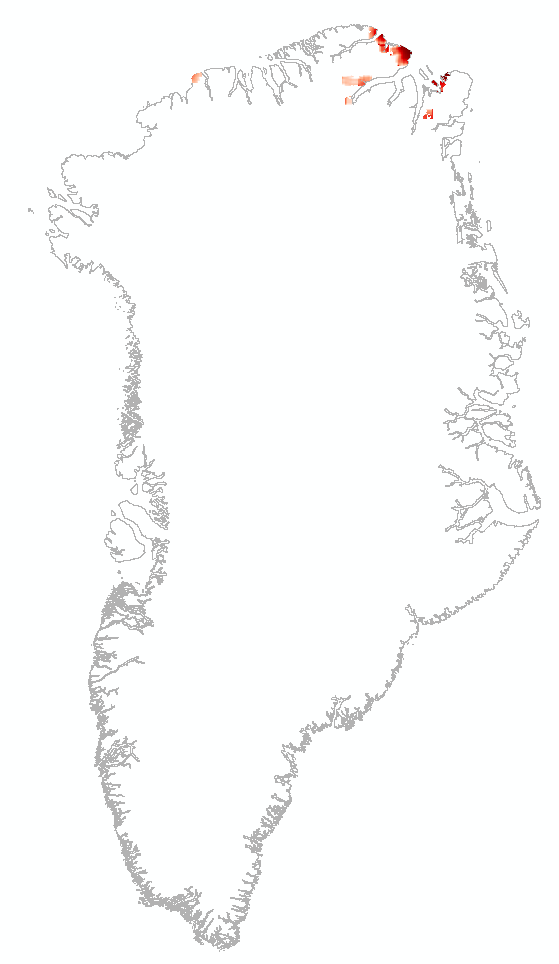

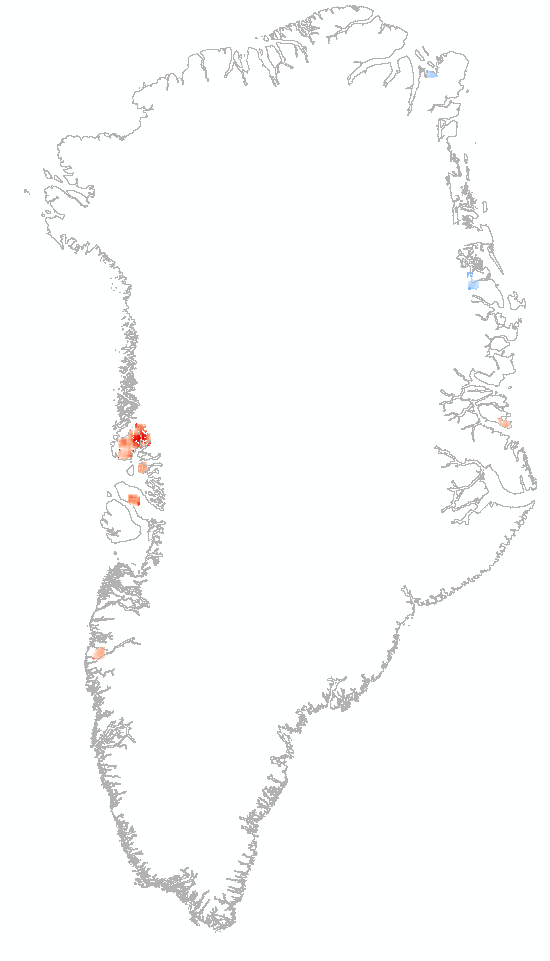

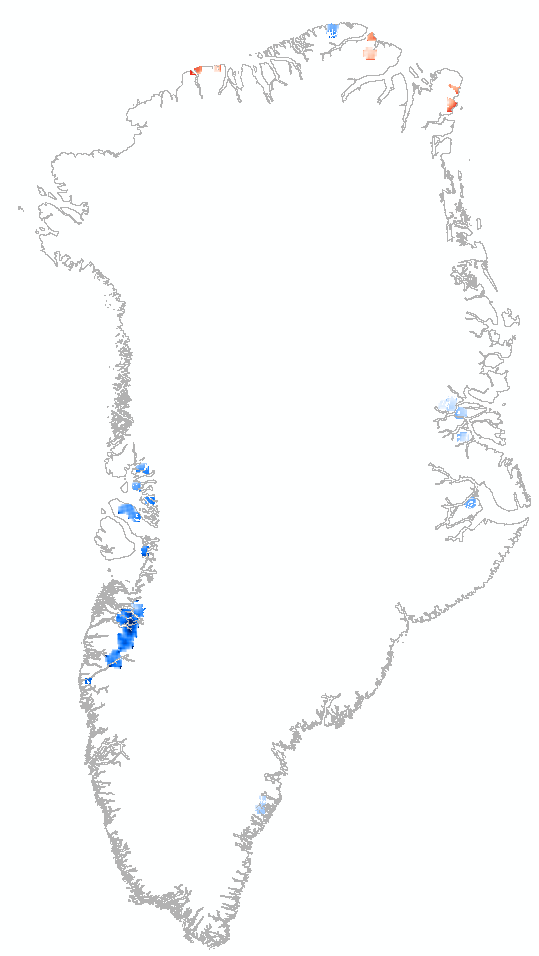

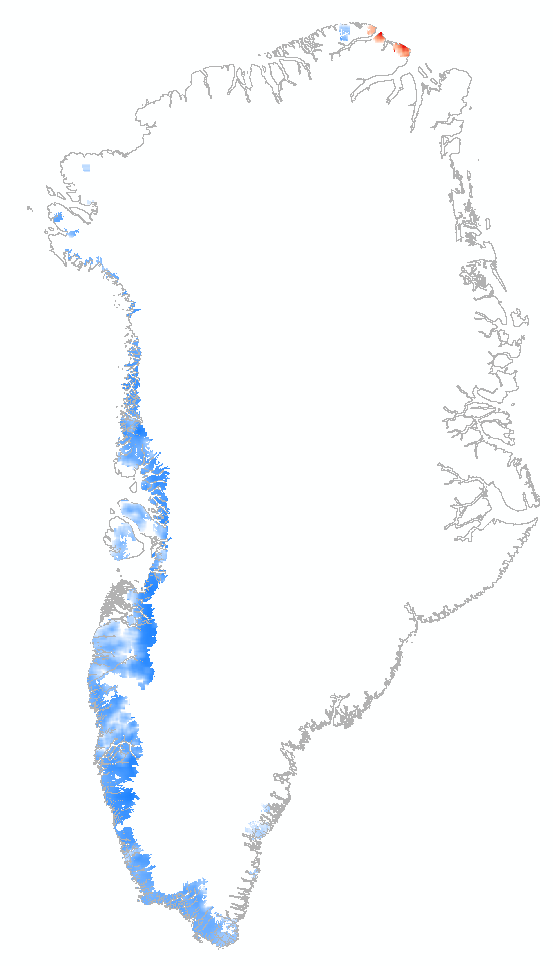


-4 -3 -2 -1 0 1 2 3 4 ⁰C

MAM JJA SON DJF


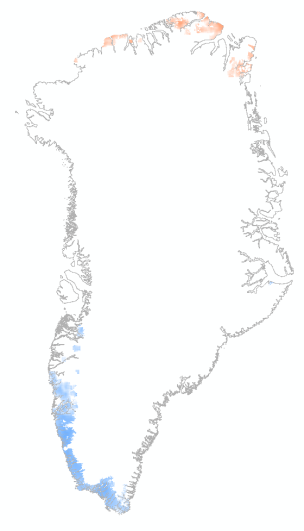


Annual

Supporting figure S5: Significant changes from 2001-2015 based on MAR v3.5.2 at 5km, forced with ERA Interim. Trends are analyzed with seasonal Mann Kendall and Sen slope. The general picture is consistent with MODIS data, i.e. only few significant changes on an annual timescale (<0.17 degrees change per year). Only significant (p < 0.05) trends in air temperature during 2001-2015 with more than 80 significant pixels within 20x20 km are plotted. See Fig. 4S for the climate normal period 1986-2015. The maps are made using ArcMap 10.3.


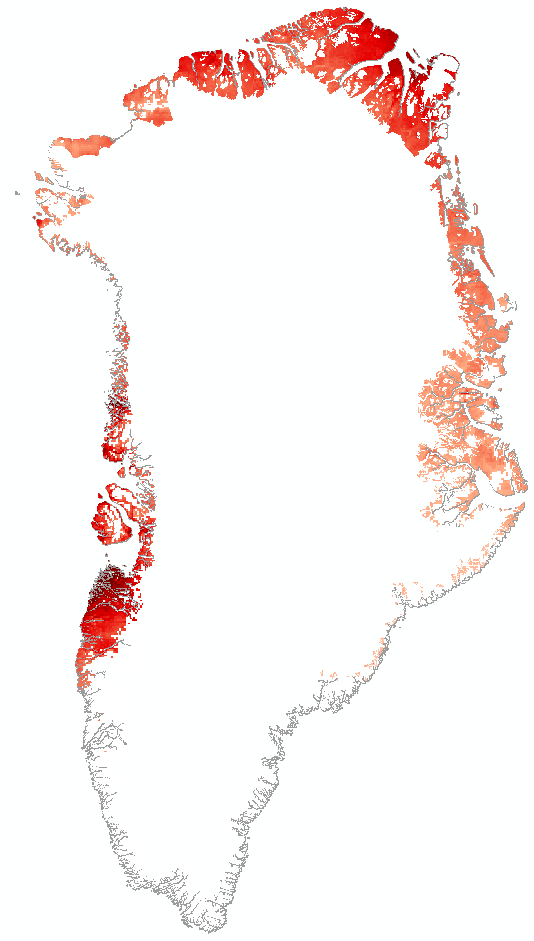

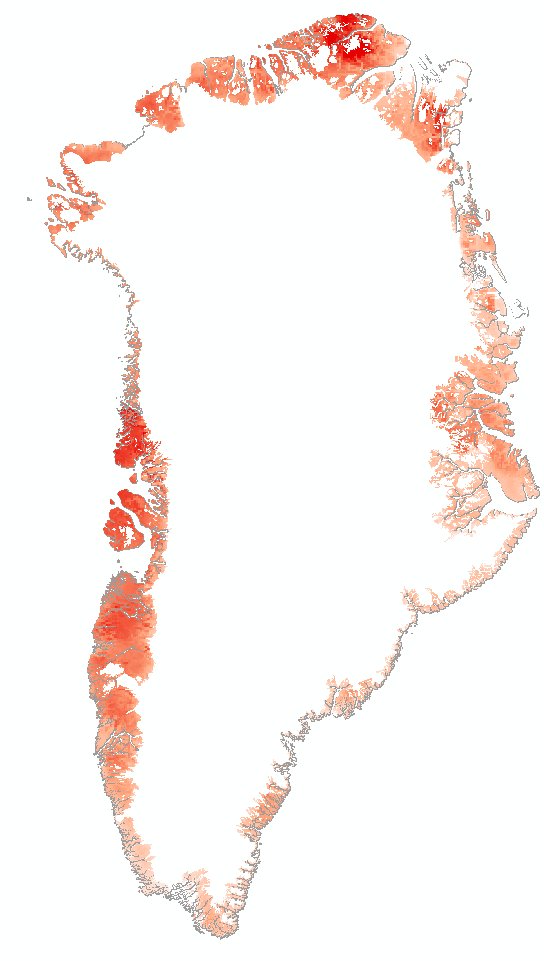

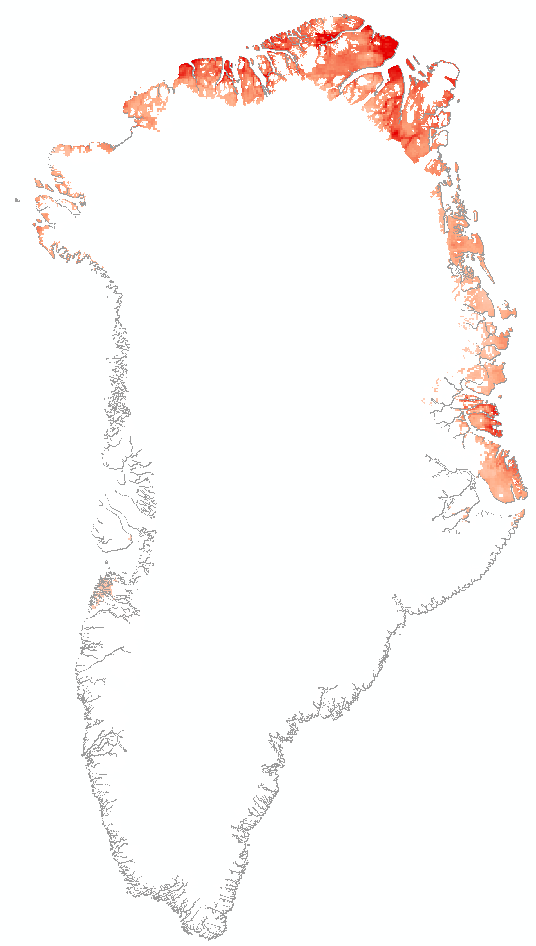

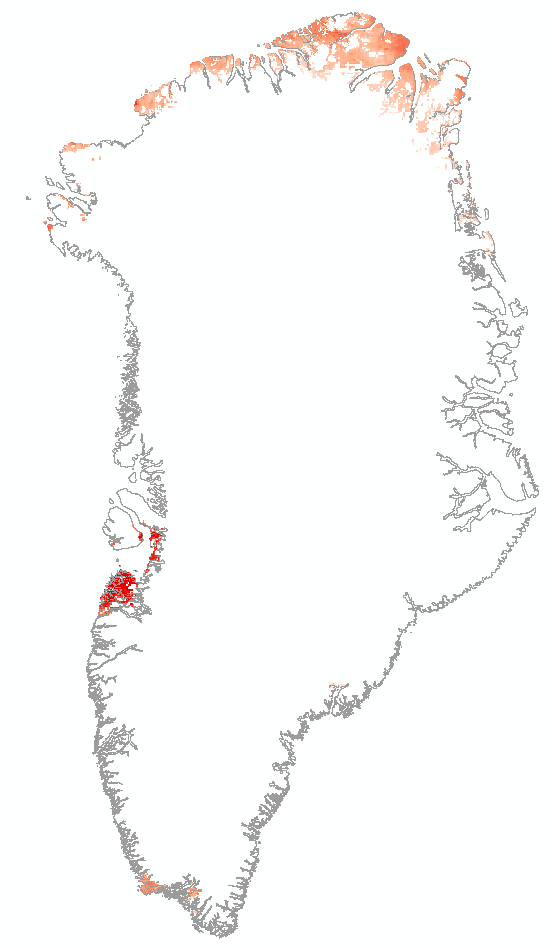


MAM JJA SON DJF


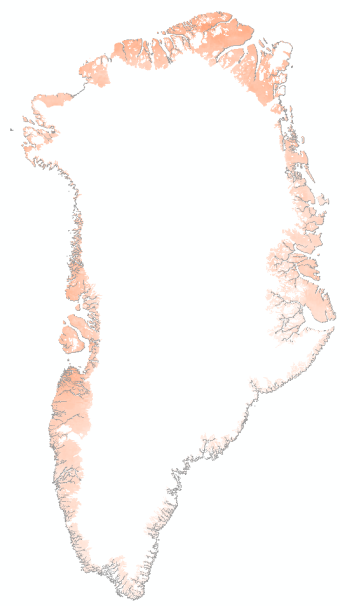


-4 -3 -2 -1 0 1 2 3 4 ⁰C

Annual

Supporting figure S6: Significant changes from 1986-2015 based on MAR v3.5.2 at 5km, forced with ERA Interim. Trends are analyzed with seasonal Mann Kendall and Sen slope. Only significant (p < 0.05) trends in air temperature during 2001-2015 with more than 80 significant pixels within 20x20 km are plotted. The maps are made using ArcMap 10.3.


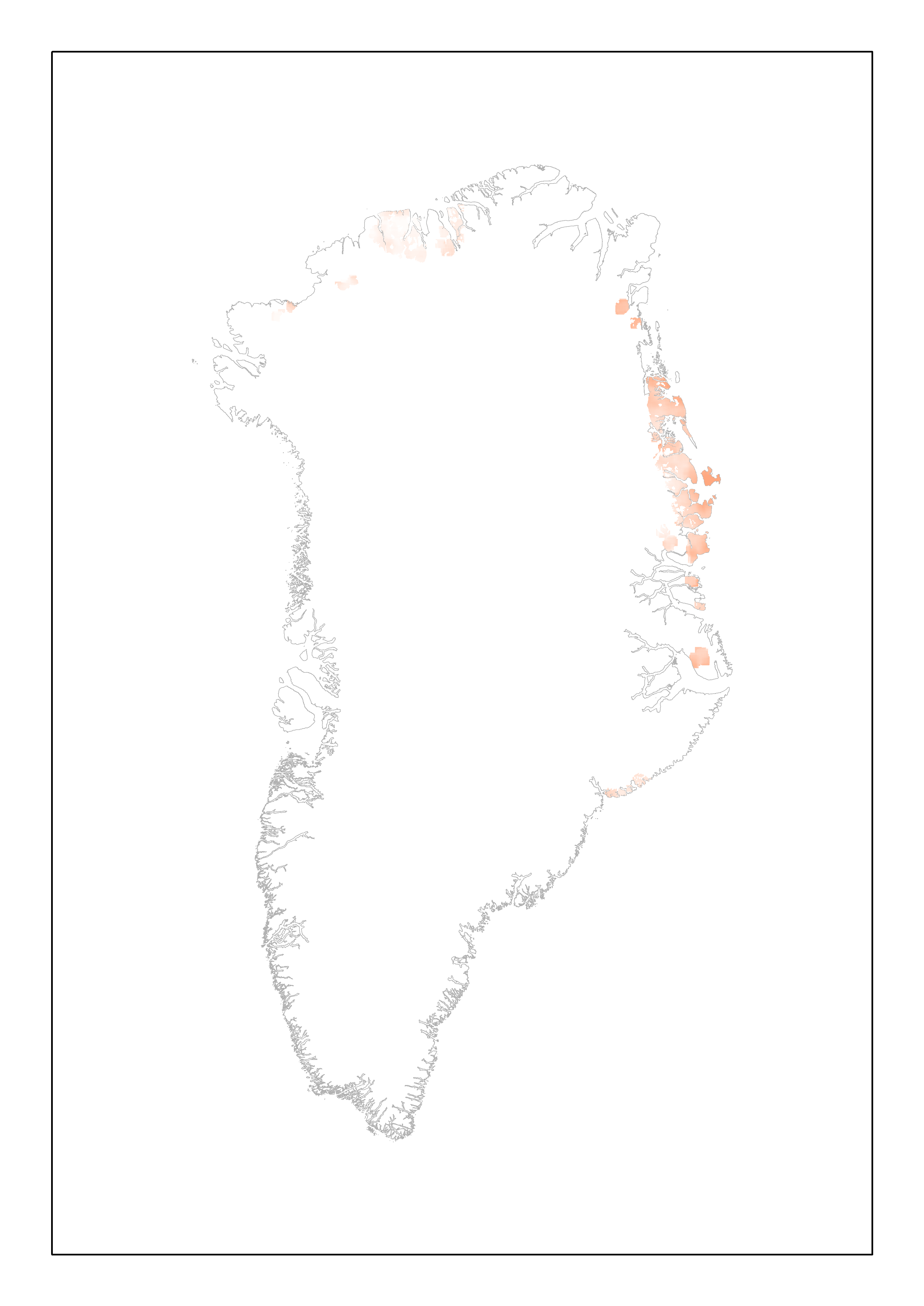

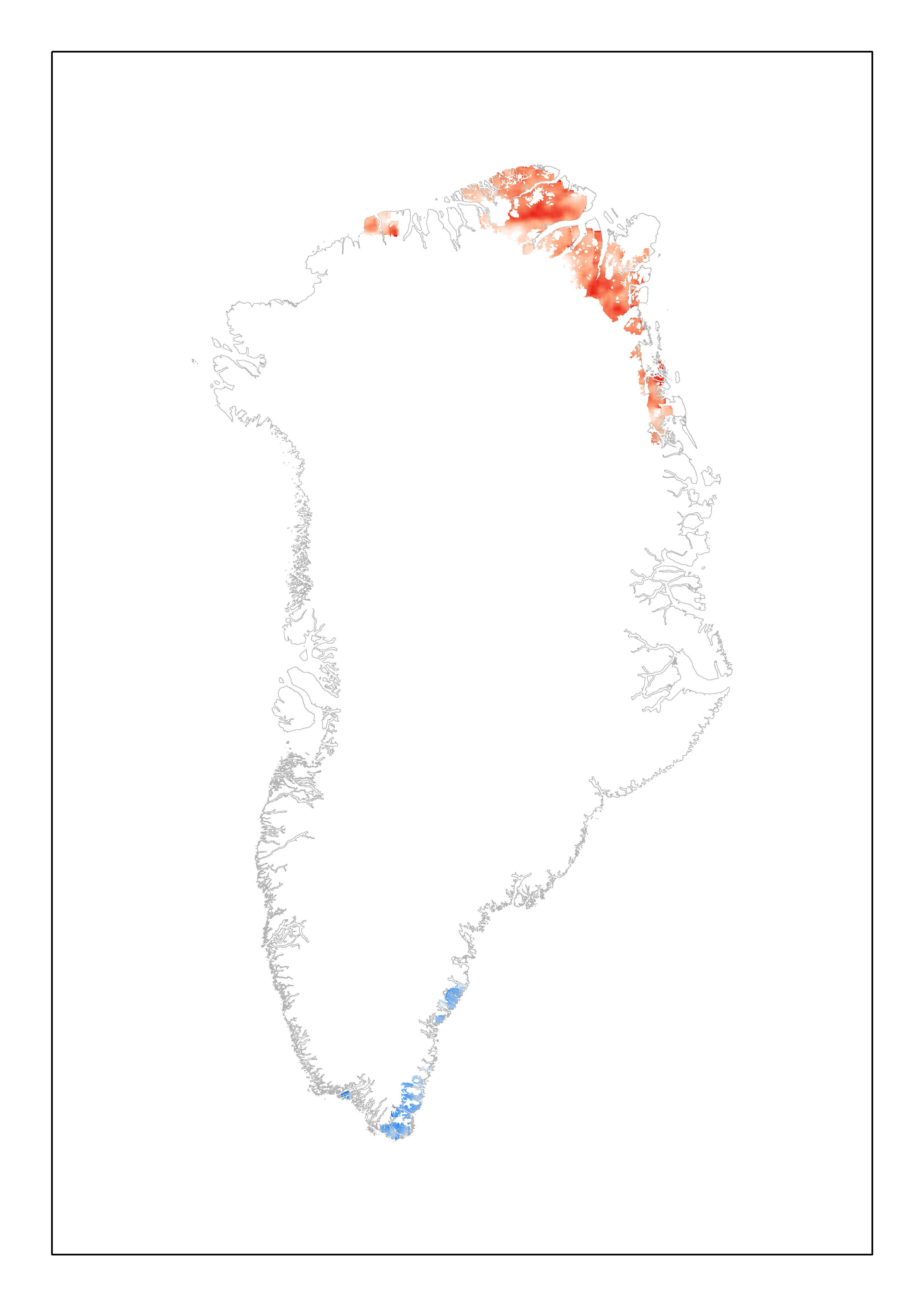

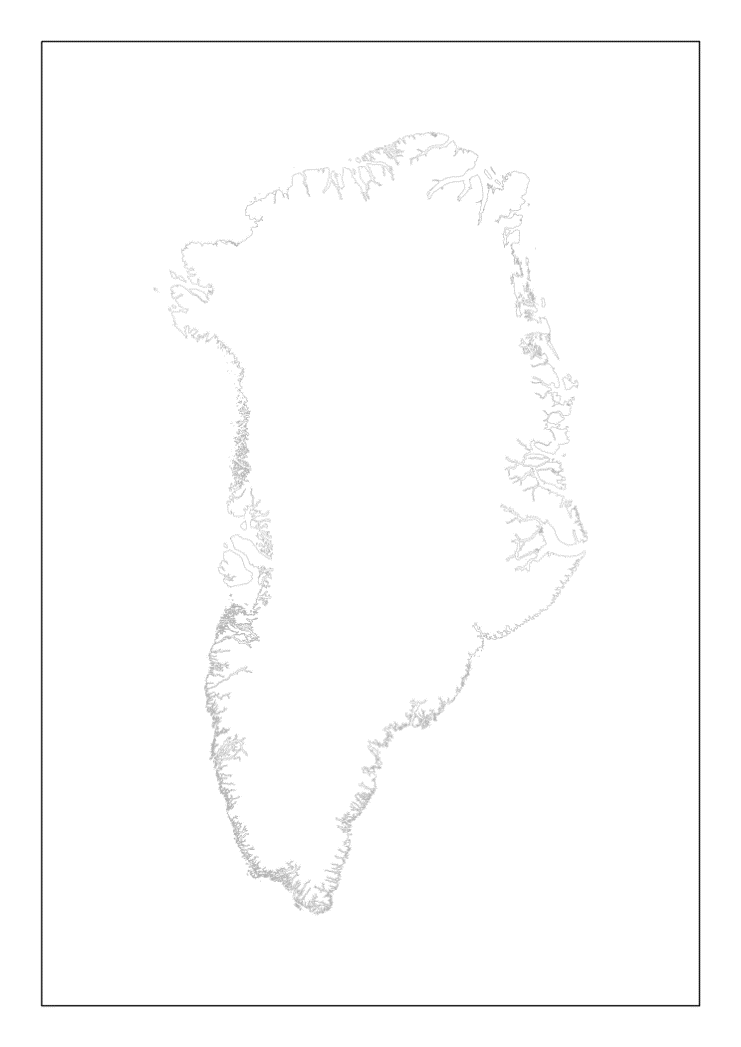

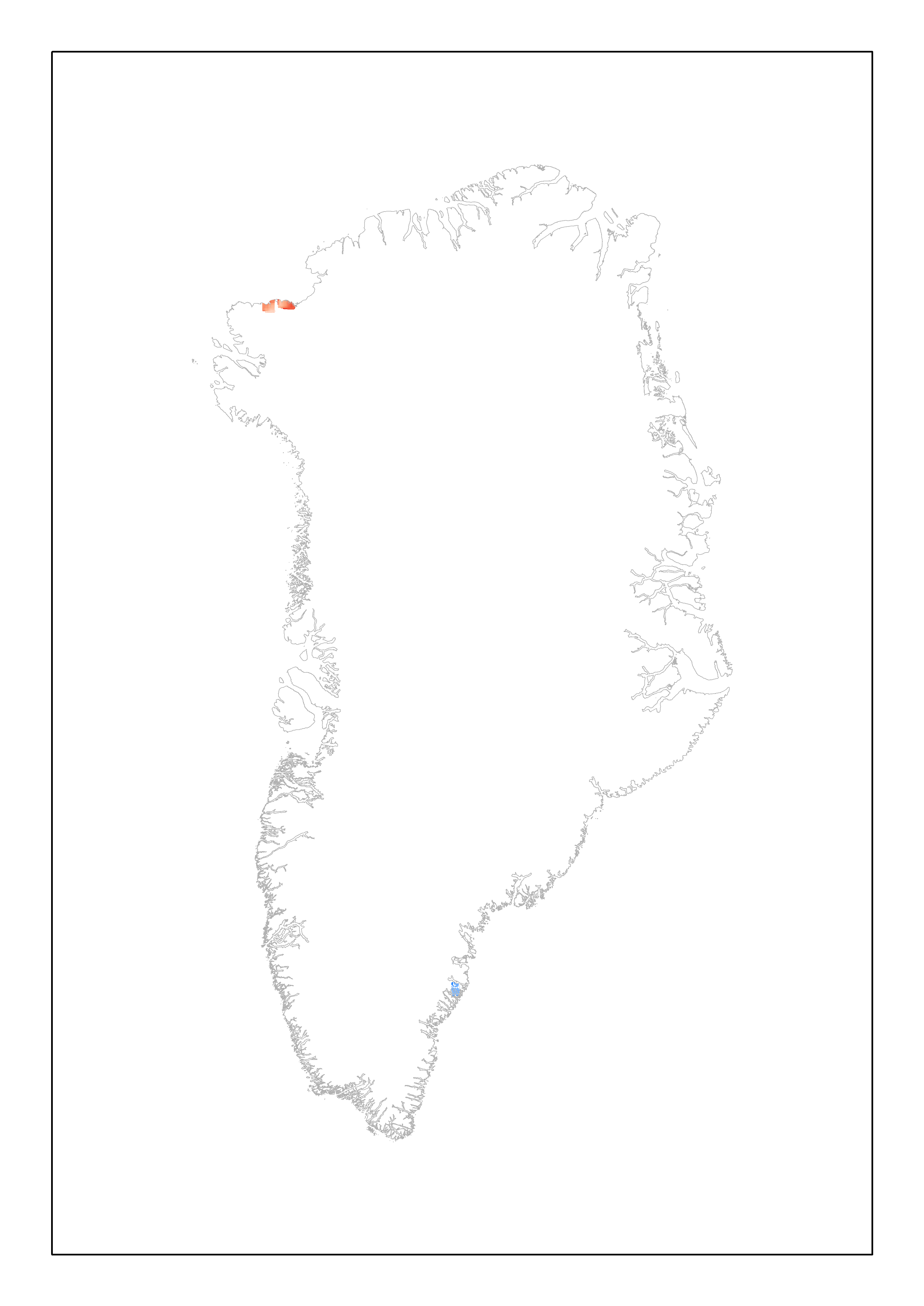

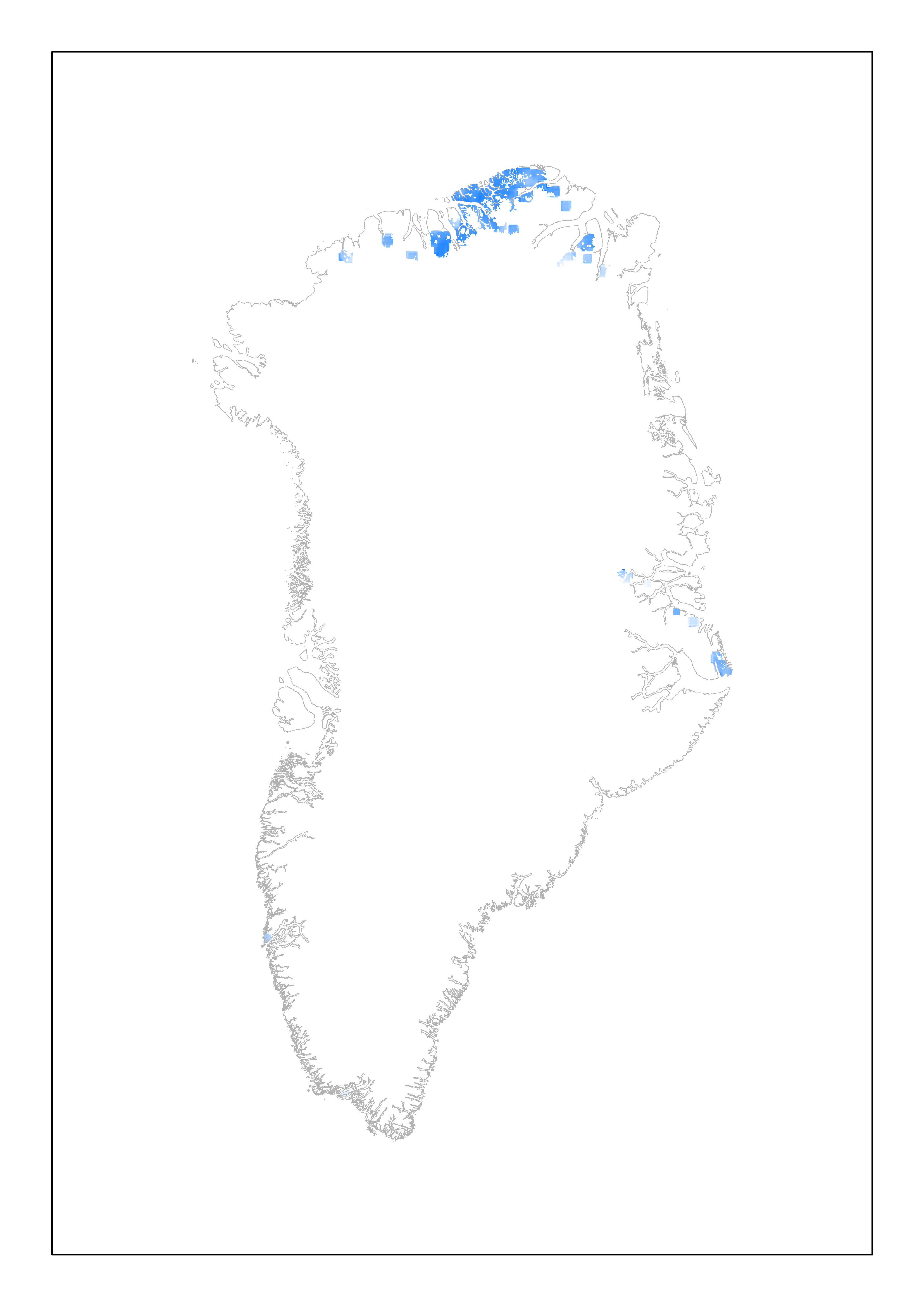

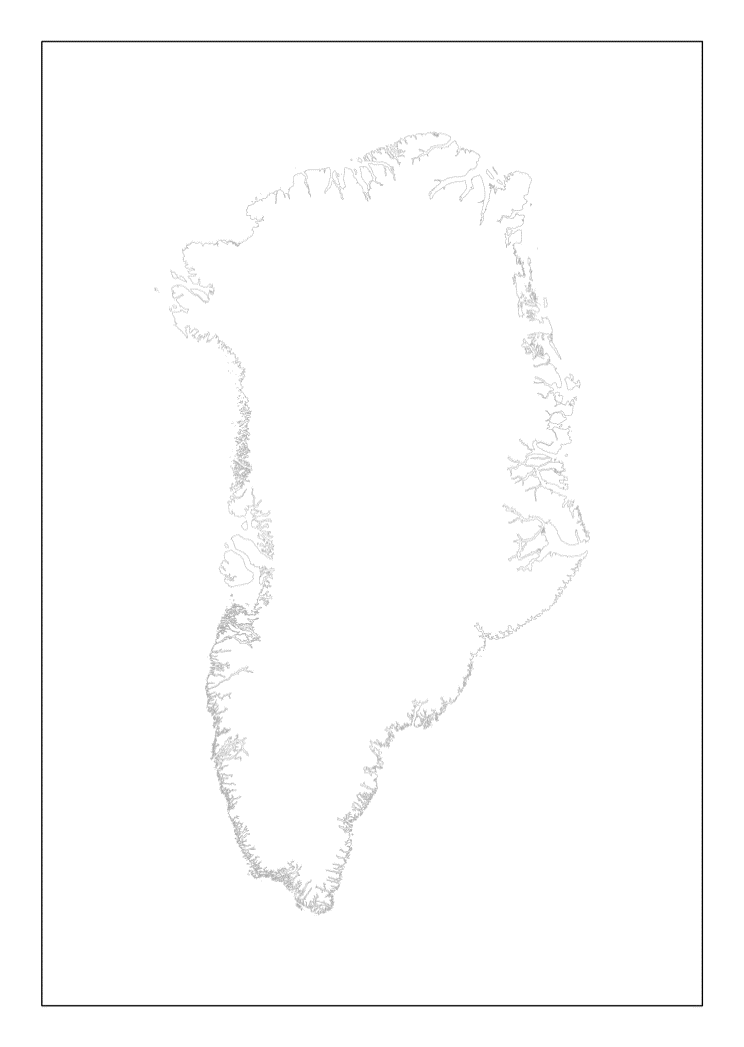
Supporting figure S7A: Significant trends (p < 0.05) in air temperature equivalents from gap-filled MODIS LST 2001-2015. The data has been filtered to include only areas of 20 x 20 km with more than 80 significant pixels. The maps are made using ArcMap 10.3.

Jan Feb Mar

Apr May Jun

-6 -4 -2 -1 0 1 2 4 6 ⁰C

Supporting figure S7B: Significant trends (p < 0.05) in air temperature equivalents from gap-filled MODIS LST 2001-2015. The data has been filtered to include only areas of 20 x 20 km with more than 80 significant pixels.
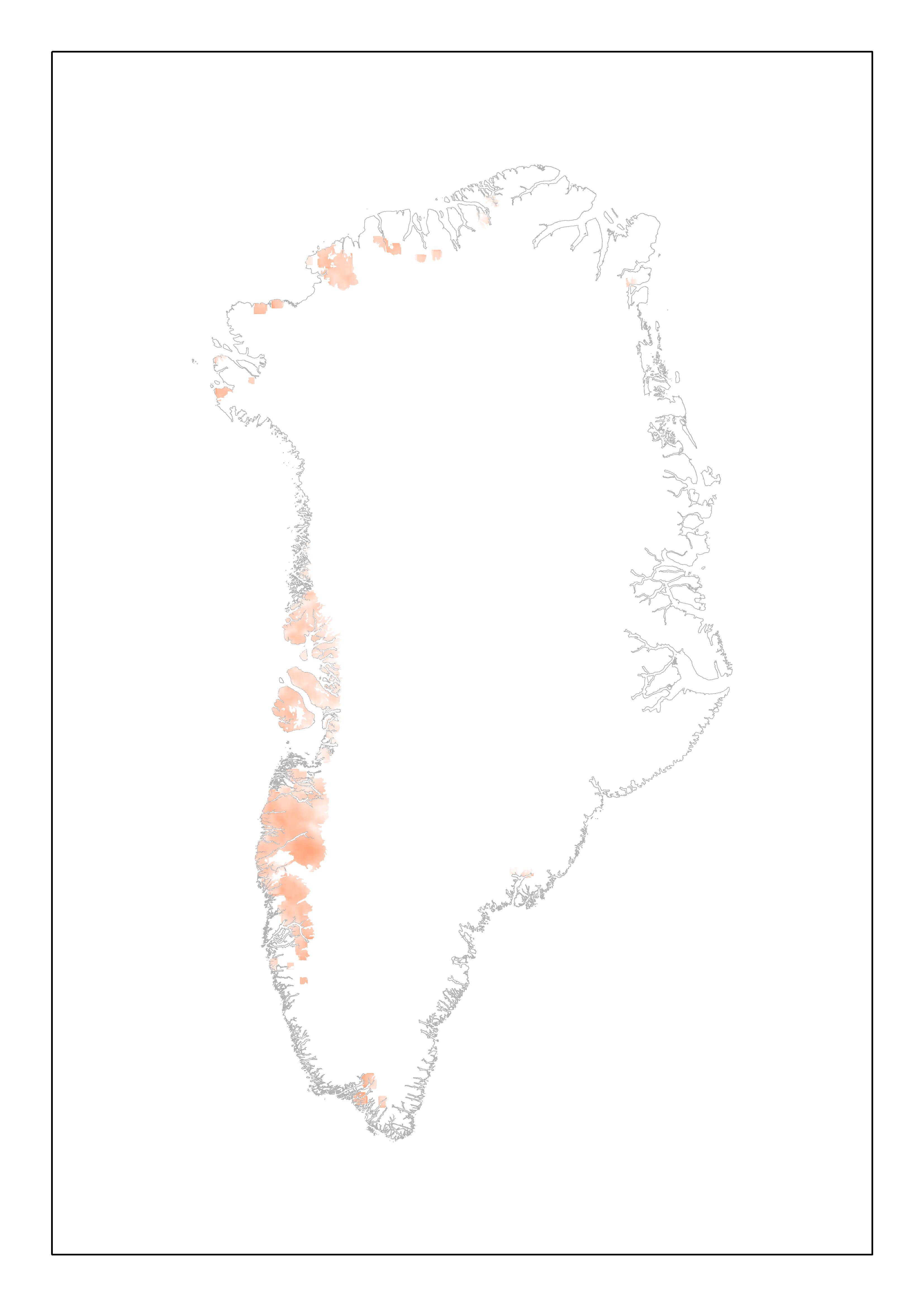

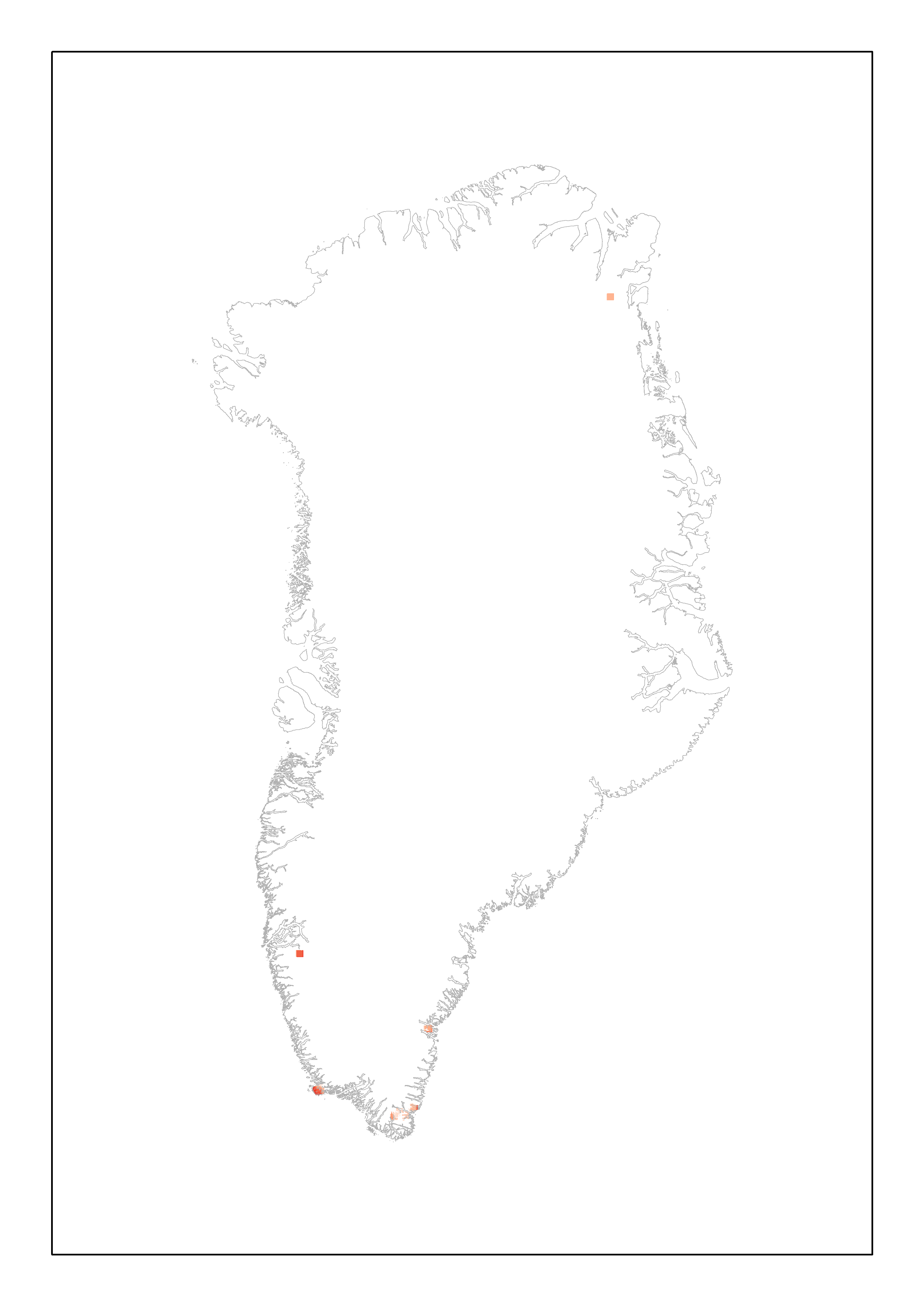

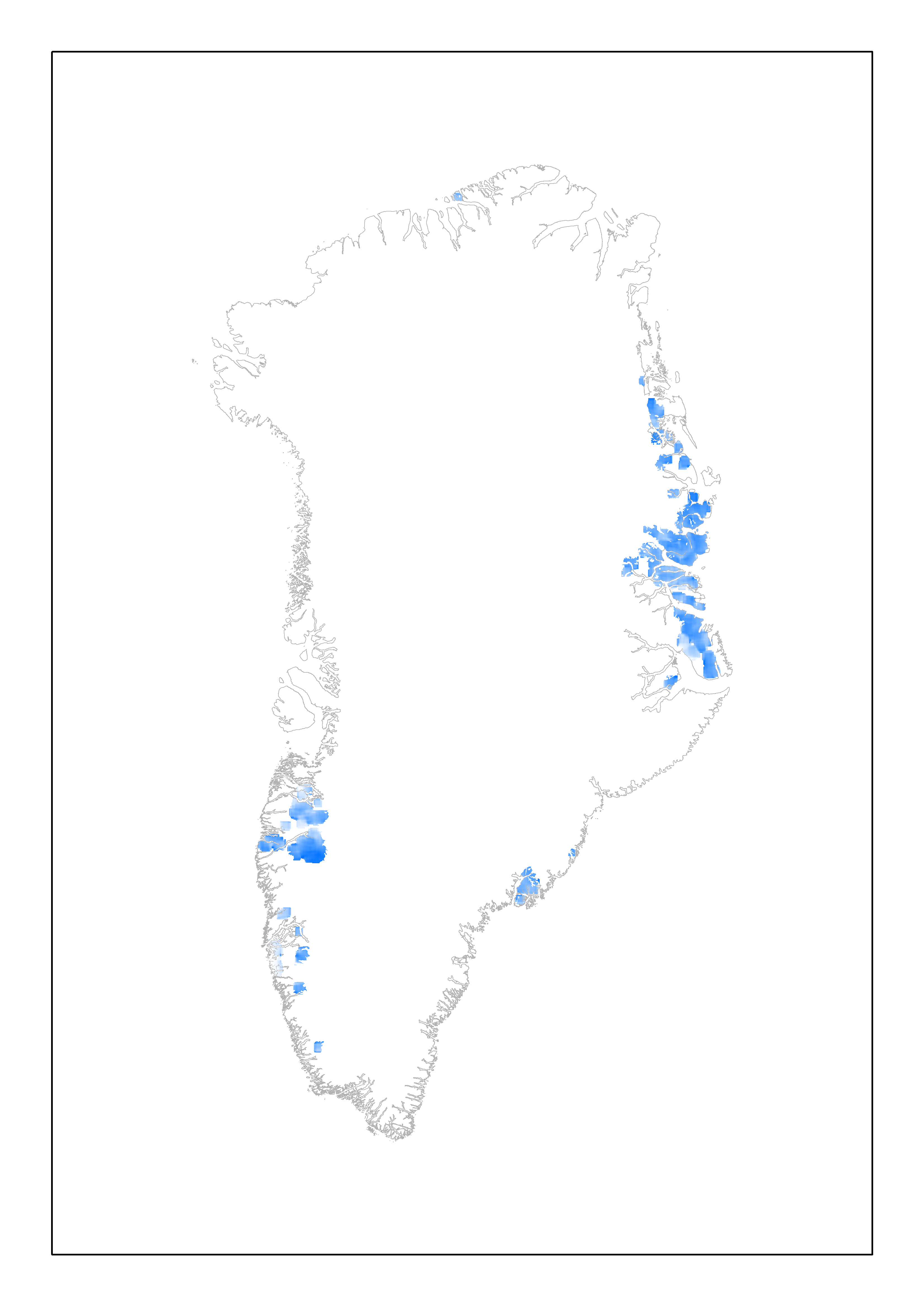

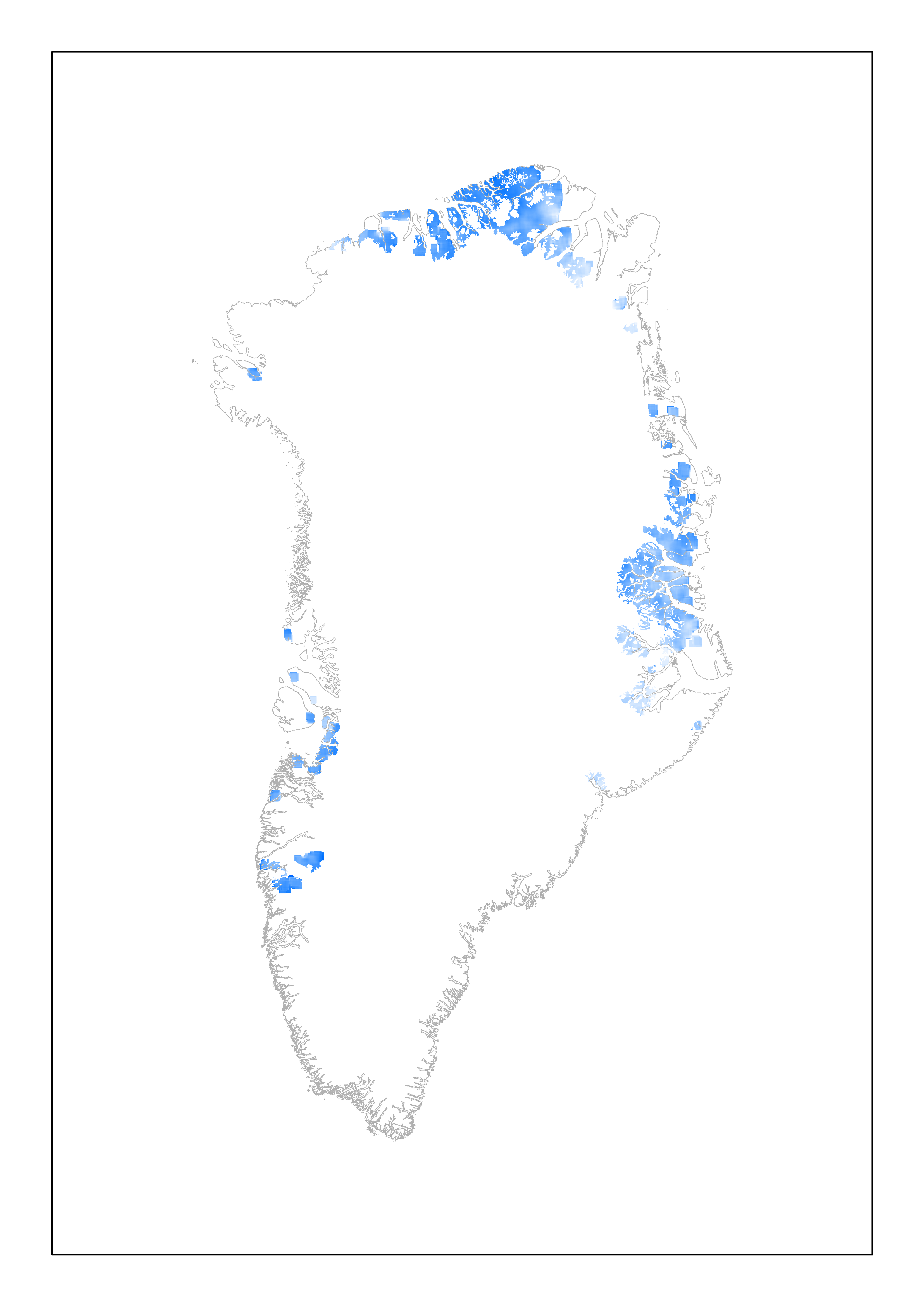

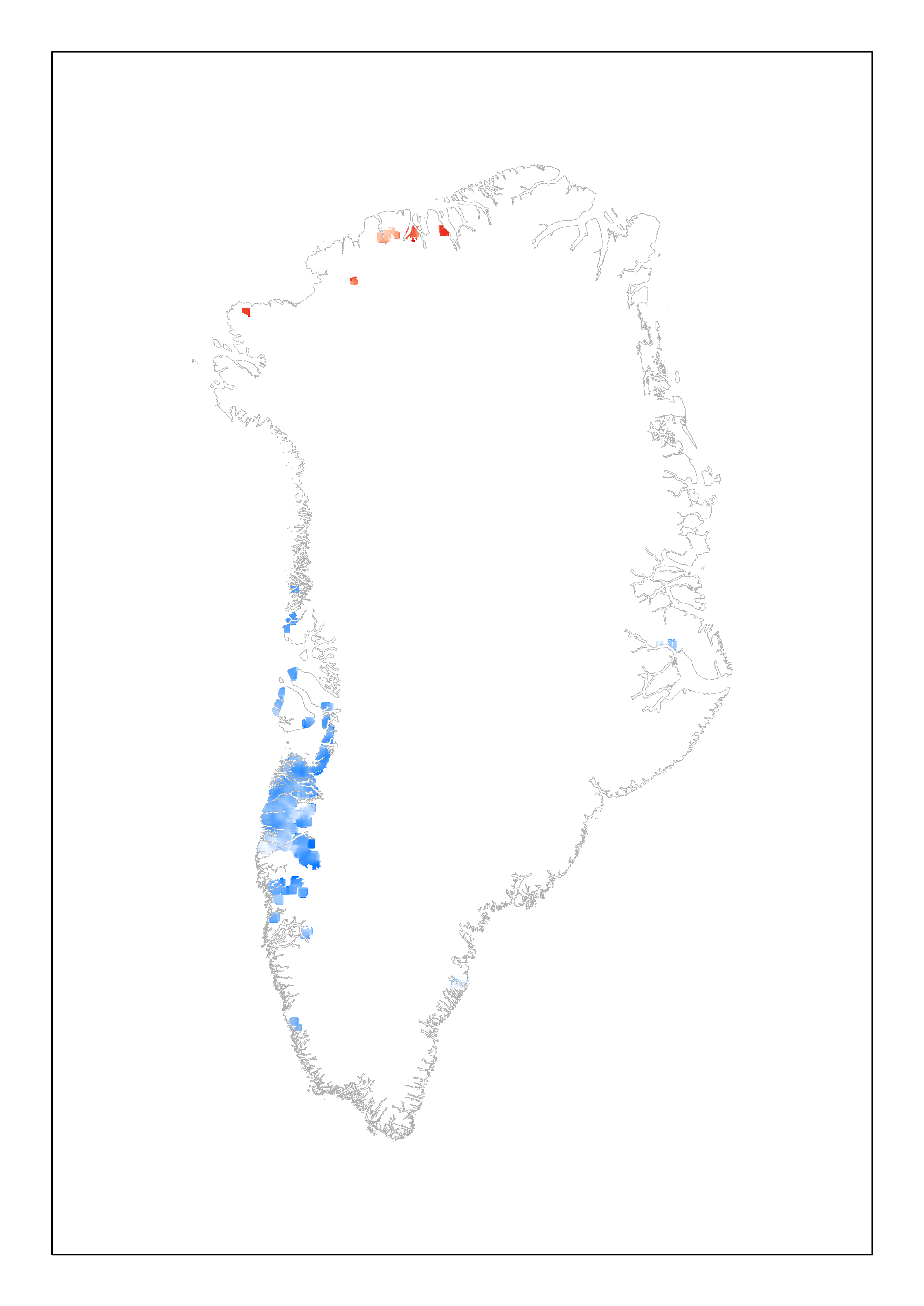

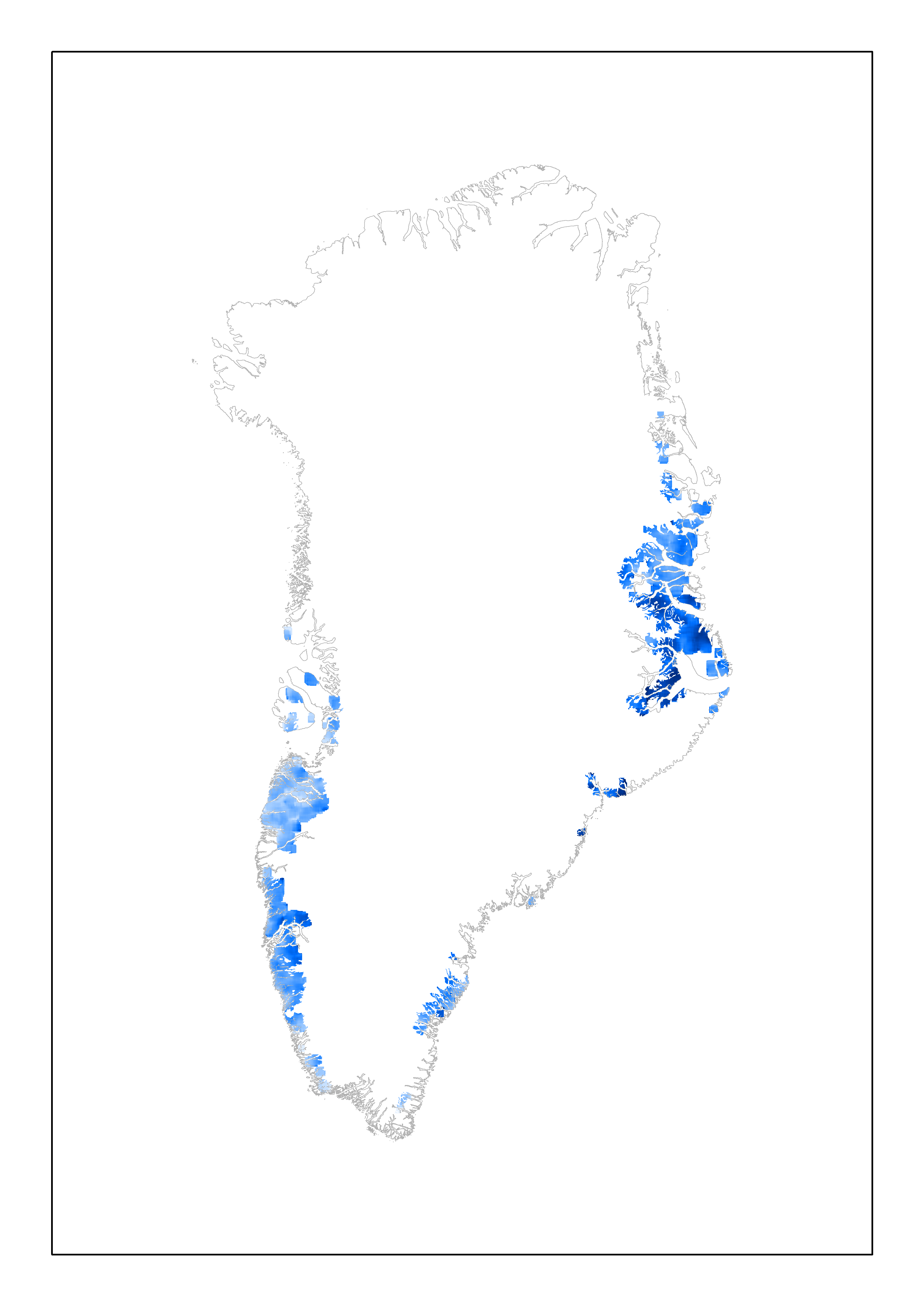
 The maps are made using ArcMap 10.3.

Jul Aug Sep

Oct Nov Dec

-6 -4 -2 -1 0 1 2 4 6 ⁰C


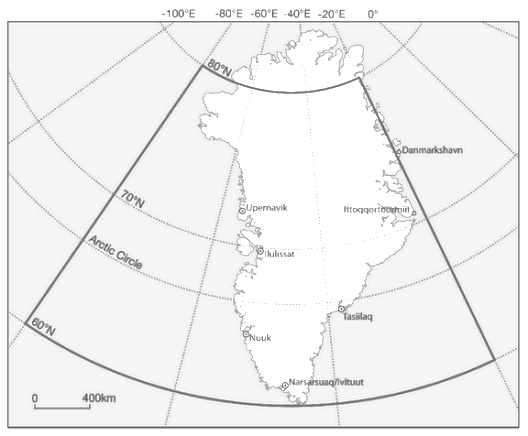

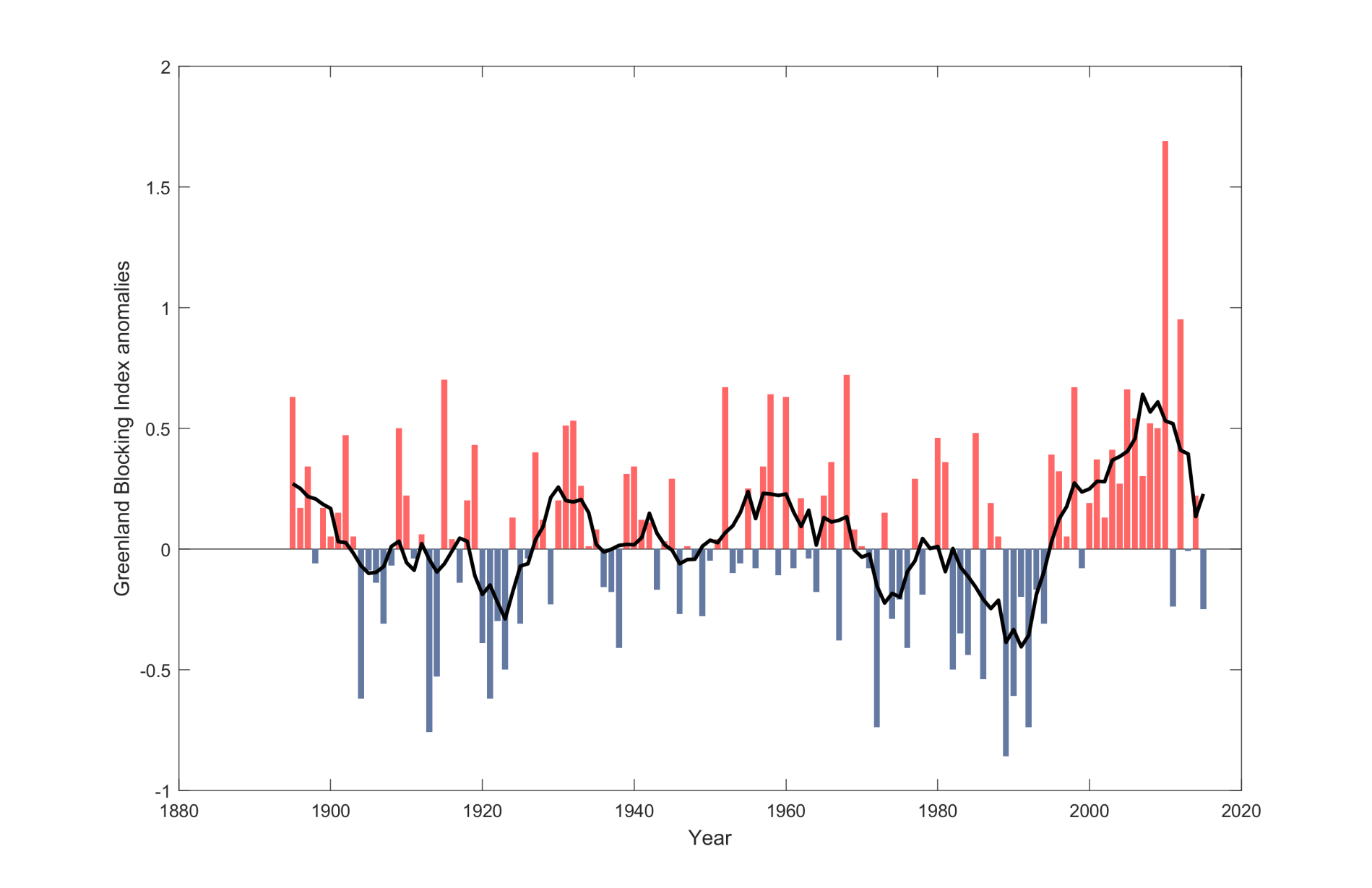


Supporting figure S8: Greenland Blocking Index (GBI) anomalies from 1895 to 2015, based on the mean 500 hPa geopotential height for the 60-80 ^o^N and 20-80 ^o^W region, see spatial domain in the upper left corner of the panel^18^. The black line illustrates a 7 point running average. The map is made using ArcMap 10.3.

Supporting table S1: Results and t-tests of correlation analyses (ordinary least squares) between the Greenland Blocking Index (GBI) and measured 2m air temperatures. The analyses were made for Tasilaq (east coast of Greenland) as well as Nuuk and Ilulissat (west coast of Greenland).

| **Period/Location** | **Nuuk** | **Ilulissat** | **Tasilaq** |
| --- | --- | --- | --- |
| **MAM** | P val < 0.01 R sq = 0.48 Slope = 1.85 | P val < 0.01 R sq = 0.24 Slope = 1.92 | P val = 0.04 R sq = 0.03 Slope = 0.45 |
| **JJA** | P val < 0.01 R sq = 0.31 Slope = 0.79 | P val < 0.01 R sq = 0.23 Slope = 0.66 | P val = 0.02 R sq = 0.05 Slope = 0.28 |
| **SON** | P val < 0.01 R sq = 0.34 Slope = 1.02 | P val < 0.01 R sq = 0.34 Slope = 1.29 | P val < 0.01 R sq = 0.11 Slope = 0.71 |
| **DJF** | P val < 0.01 R sq = 0.61 Slope = 2.97 | P val < 0.01 R sq = 0.41 Slope = 3.74 | P val = 0.09 |
| **Annual** | P val < 0.01 R sq = 0.39 Slope = 1.87 | P val < 0.01 R sq = 0.23 Slope = 1.96 | P val < 0.01 R sq = 0.12 Slope = 0.89 |
